# Impact of *c-JUN* deficiency on thalamus development in mice and human neural models

**DOI:** 10.1186/s13578-024-01303-8

**Published:** 2024-12-20

**Authors:** Jiantao Shi, Qing Chen, Jianheng Lai, Jieying Zhu, Ran Zhang, Md. Abdul Mazid, Dongwei Li, Huanxing Su, Dajiang Qin

**Affiliations:** 1 State Key Laboratory of Quality Research in Chinese Medicine, Institute of Chinese Medical Sciences, University of Macau, Macao, China; 2 Key Laboratory of Biological Targeting Diagnosis, Therapy and Rehabilitation of Guangdong Higher Education Institutes, The Fifth Affiliated Hospital of Guangzhou Medical University, Guangzhou, China; 3 Guangdong Engineering Technology Research Center of Biological Targeting Diagnosis, Therapy and Rehabilitation, The Fifth Affiliated Hospital, Guangzhou Medical University, Guangzhou, China; 4 CAS Key Laboratory of Regenerative Biology, Center for Cell Lineage and Development, Guangzhou Institutes of Biomedicine and Health, Chinese Academy of Sciences, Guangzhou, China; 5 Laboratory of Integrative Biology, Guangzhou Institutes of Biomedicine and Health, Chinese Academy of Sciences, Guangzhou, China; 6 Guangdong Engineering Research Center of Early Clinical Trials of Biotechnology Drugs, The Fifth Affiliated Hospital,, Guangzhou Medical University, Guangzhou, China; 7 Bioland Laboratory Guangzhou Regenerative Medicine and Health Guangdong Laboratory, Guangzhou, China; 8 Centre for Regenerative Medicine and Health, Hong Kong Institute of Science & Innovation, Chinese Academy of Sciences, Hong Kong SAR, China

**Keywords:** *c-JUN*, AP-1, NPC, Cerebral organoids, Thalamus organoids, Neural development

## Abstract

**Background:**

c-Jun is a key regulator of gene expression. Through the formation of homo- or heterodimers, c-JUN binds to DNA and regulates gene transcription. While *c-Jun* plays a crucial role in embryonic development, its impact on nervous system development in higher mammals, especially for some deep structures, for example, thalamus in diencephalon, remains unclear.

**Methods:**

To investigate the influence of *c-JUN* on early nervous system development, *c-Jun* knockout (KO) mice and *c-JUN* KO H1 embryonic stem cells (ESCs)-derived neural progenitor cells (NPCs), cerebral organoids (COs), and thalamus organoids (ThOs) models were used. We detected the dysplasia via histological examination and immunofluorescence staining, omics analysis, and loss/gain of function analysis.

**Results:**

At embryonic day 14.5, *c-Jun* knockout (KO) mice exhibited sparseness of fibers in the brain ventricular parenchyma and malformation of the thalamus in the diencephalon. The absence of *c-JUN* accelerated the induction of NPCs but impaired the extension of fibers in human neuronal cultures. COs lacking *c-JUN* displayed a robust PAX6^+^/NESTIN^+^ exterior layer but lacked a fibers-connected core. Moreover, the subcortex-like areas exhibited defective thalamus characteristics with transcription factor 7 like 2-positive cells. Notably, in guided ThOs, *c-JUN* KO led to inadequate thalamus patterning with sparse internal nerve fibers. Chromatin accessibility analysis confirmed a less accessible chromatin state in genes related to the thalamus. Overexpression of *c-JUN* rescued these defects. RNA-seq identified 18 significantly down-regulated genes including RSPO2, WNT8B, MXRA5, HSPG2 and PLAGL1 while 24 genes including MSX1, CYP1B1, LMX1B, NQO1 and COL2A1 were significantly up-regulated.

**Conclusion:**

Our findings from in vivo and in vitro experiments indicate that *c-JUN* depletion impedes the extension of nerve fibers and renders the thalamus susceptible to dysplasia during early mouse embryonic development and human ThO patterning. Our work provides evidence for the first time that *c-JUN* is a key transcription regulator that play important roles in the thalamus/diencephalon development.

**Supplementary Information:**

The online version contains supplementary material available at 10.1186/s13578-024-01303-8.

## Introduction

The proto-oncogene *c-Jun* is considered a homolog of *v-Jun*, identified in transformed cells carrying the genome of replication-defective avian sarcoma virus 17 [1, 2]. It is the most extensively studied protein within the activator protein-1 (AP-1) family. AP-1 refers to dimers formed by proteins of the JUN (c-JUN, JUNB, and JUND), Fos, and activating transcription factor families [3, 4], playing pivotal roles in various cellular processes such as proliferation, apoptosis, survival, tumorigenesis, and tissue morphogenesis [5].

Through homodimer or heterodimer formation, c-JUN binds to DNA and regulates gene transcription. c-JUN possesses at least five phosphorylation sites, three of which are located upstream of the base region of the DNA-binding domain (residues between 227 and 252), and two within the N-terminal transcriptional activation domain at serines 63 and 73 [6, 7]. Extracellular signals induce post-translational modifications of c-JUN, altering its transcriptional activity and target gene expression. This intricate regulatory network enables c-JUN to cross-talk, amplify, and integrate different signals for tissue development and disease. Notably, the *c-Jun* promoter contains binding sites for nuclear factor kappa-light-chain-enhancer of activated B cells, SP1, and CCAAT-binding transcription factors, and notably, a high-affinity AP-1 binding site [8, 9]. Activation of AP-1 stimulates the *c-Jun* promoter and gene expression, with *c-Jun* expression further enhancing AP-1, thereby promoting its gene promoter activation [8, 10]. This autocrine and feed-forward mechanism not only prolongs AP-1 activity but also amplifies it, enabling *c-Jun* to efficiently convert transient biochemical signals into sustained AP-1 activity, acting as a potent transcription factor to regulate long-term biological outcomes.

In studies of somatic cell reprogramming, *c-Jun* has emerged as a guardian of somatic cell fate, with its inhibition facilitating pluripotency [11]. A recent study revealed that *c-Jun* knockout improved cardiomyocyte generation from human pluripotent stem cells, suggesting that *c-Jun* modulates cardiomyocyte fate by regulating H3K4me3 modification and chromatin accessibility [12].

Within the nervous system, *c-Jun* participates in the anti-neuralization activity of bone morphogenetic protein 4 (BMP4) in *Xenopus laevis* neurogenesis. It synergistically inhibits neuronal development of African *Xenopus laevis* ectoderm with BMP4 signaling [13]. Moreover, an interaction between *Trim69* and *c-Jun* has been identified in zebrafish neurogenesis, with their joint knockdown rescuing deformed brains characterized by marked apoptosis of head cells and reduced expression of neuronal differentiation and stem cell markers [14]. *c-Jun* and BH3 factor inhibition aid *Dbx1* in the prevention of apoptotic cell death in the inferior colliculus at the top of the midbrain during mouse postnatal life [15].

After nearly three decades of extensive research, *c-Jun* remains at the center of a molecular network with enigmatic functional properties, some of which remain to be fully elucidated. Building upon the aforementioned studies and considering the wide distribution and relatively high expression of *c-Jun* in the central nervous system (CNS) of both embryonic and adult mouse brains [16, 17], we hypothesize that *c-Jun* plays significant roles in nervous system development.

In the present study, we investigated the effects of *c-Jun* knockout (KO) on neural development using both in vivo (mouse model) and in vitro (2D neural progenitor cells [NPCs], 3D human cerebral organoids [COs], and human thalamus organoids [ThOs]) approaches. In the in vivo model, *c-Jun* KO mouse embryos at E14.5 exhibited a normal cortical structure but showed relatively loosened intercellular connections and sparse fibers in the ventricular parenchyma. The profile of the thalamus/diencephalon was abnormal, with disrupted expression patterns of the thalamus-specific marker protein transcription factor 7 like 2 (TCF7L2) observed within affected embryos. In the in vitro model, *c-JUN* KO promoted the differentiation of human embryonic stem cells (ESCs) into NPCs but weakened fiber extension and adhesion ability in neuronal culture. In the unguided CO model, *c-JUN* KO resulted in whole-brain analogs prone to form more robust cortex-like structures but with a relatively loose interior. A range of genes associated with cortex development were upregulated, whereas those related to intercellular adhesion were significantly downregulated. In the guided ThOs, thalamus analogs patterning was significantly hindered in the KO group, and these defects could be rescued by overexpression (OE) of *c-JUN*. An assay for transposase-accessible chromatin with high-throughput sequencing (ATAC-seq) analysis confirmed a less accessible chromatin state in thalamus-related genes compared to the control. RNA seq revealed significantly altered genes and pathways.

## Results

### *c-Jun* KO mouse embryos exhibited a loss of fibers in the ventricular parenchyma and malformation of the thalamus in the diencephalon

Initially, we crossed heterozygous parents and collected mouse embryos ranging from embryonic day E11.5 to E14.5 (Table S1). After fixation, we recorded morphological features and performed genotyping. Subsequently, we sectioned the embedded embryos for hematoxylin and eosin (H&E) staining or immunofluorescence (IF) analysis. In our observation, the phenotypes of *c-Jun*^*+/+*^ and *c-Jun*^*+/−*^ embryos were essentially identical. Among embryos at E11.5–E12.5, which exhibited normal size, no significant differences were observed in overall appearance or H&E staining (data not shown). However, embryos at E13.5–14.5 displayed distinct signs in *c-Jun*^*−/−*^ embryos compared to their heterozygous or homozygous counterparts (*c-Jun*^*+/+*^, *c-Jun*^*+/−*^). These signs included diminished and discolored primary hepatic and heart regions (which was similar to previous reports) [18, 19], decreased transparency, increased whiteness of the brain region, and fading of surface blood vessels in some samples under transmitted bright light (Fig. S1A). Additionally, we occasionally observed smaller, white embryos, which appeared fragile, pale, and bloodless (Fig. S1A, H0150-8), consistent with previous reports of *c-Jun* KO embryos that ceased development and died [19, 20]. Genotyping confirmed these embryos as KO types, as expected, although their placentas appeared normal.

**Fig. 1:**
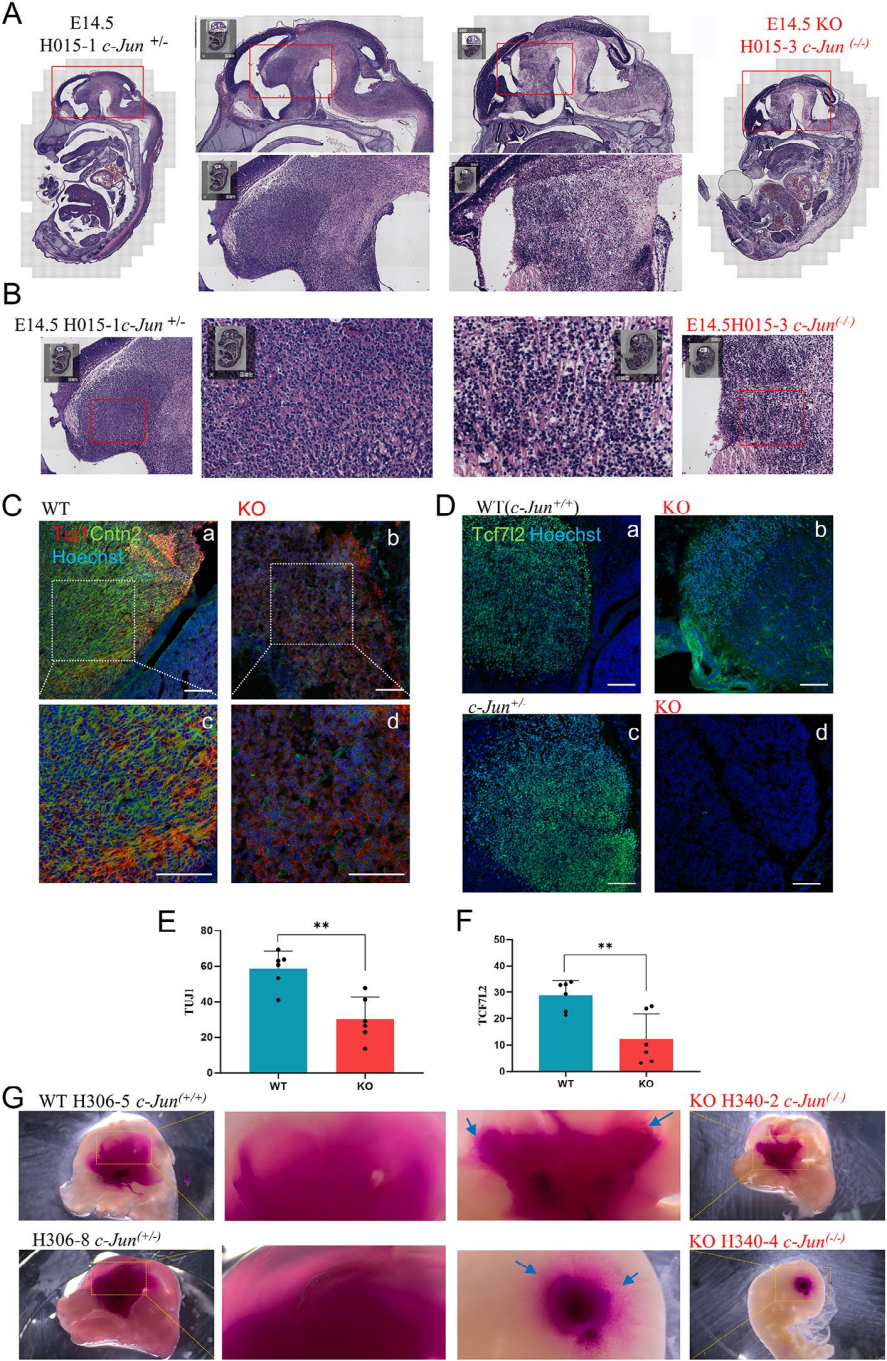
KO mouse embryos at E14.5 exhibited thalamic malformation in the diencephalon. (**A**, **B**) H&E staining of cJun^*+/-*^ and KO embryos at E14.5 revealed sparse fibers in the ventricular parenchyma and malformation of the thalamus in the diencephalon of the KO embryo. The small inset in the upper-left corner of the image displays the global view of the embryo. H015-1: “H015” was the code number of the mother mouse, and “-1” was the embryo number in the litter, hereinafter the same. (**C**) IF staining of TUJ1 and CNTN2 showed that WT had normal nerve fibers in the thalamus/diencephalon region, while those of KO were broken down with scattered distribution. a–d, Scale bar: 100 μm. (**D**) IF staining of TCF7L2 showed strong signals in the thalamus/diencephalon region of WT (a, c), while it was significantly decreased (b) or undetectable (d) in the corresponding areas in the KO groups. Scale bar: 100 μm. (**E**, **F**) Statistical analysis of IF staining of TUJ1 and TCF7L2 in the thalamus/diencephalon region of mice embryo section at E14.5. The Y-axis represents the mean gray value of signals. *n* = 6, the values are presented as the mean ± SD. *p < 0.05, **p < 0.01, ***p < 0.001. (**G**) DiI dye diffusion in the adjacent areas of the diencephalon. The middle image shows a local magnification of the region near the thalamus. The arrows indicate the areas of apparent dye penetration obstruction in KO. The embryo H340-4 *c-Jun*^*-/-*^ at the bottom-right corner was the “small and white” embryo, which was difficult to cut, thus the DiI crystals were implanted into the intact brain parenchyma area through an incision

H&E staining analysis of sagittal sections of the CNS in E14.5 embryos revealed no significant differences in the cortex between WT and KO embryos (Fig. S1D-F). However, a noticeable decrease in cell density and sparse or loose nerve fibers was observed in the brain parenchyma of KO embryos (Fig. 1A, B, Fig. S1D-F). Particularly in the diencephalon region, WT embryos exhibited a normally developed thalamus with dense cells forming a heart-shaped outline (Fig. 1A, H015-1 c*-Jun*^*+/-*^) [21], Whereas, KO embryos from the same litter displayed a malformed thalamus (Fig. 1A, H015-3 c-*Jun*^*−/−*^). Similar malformations were observed in other litters (Fig. S1D-F). IF analysis further revealed that WT embryos had normal TUJ1^+^, CNTN2^+^ nerve fibers in the thalamus/diencephalon region, whereas these were significantly reduced in KO embryos (Fig. 1C). Staining for the thalamic characteristic marker TCF7L2 showed strong signals in WT embryos (Fig. 1D, a, c), but significant decreases (Fig. 1D, b) or undetectable levels (Fig. 1D, d) in KO embryos. IF staining revealed significantly reduced TUJ1 and TCF7L2 signals in KO embryos (Fig. 1E, F). To further verify nerve fiber loss, DiI (1, 1’-dioctadecyl-3, 3, 3’, 3’-tetramethylindocarbocyanine perchlorate) dye was used to examine dye diffusion along nerve fibers. In WT groups, DiI dye diffused smoothly along the mesencephalon and telencephalon (Fig. 1G). However, in KO groups, the dye gathered in the thalamic region with a truncated diffusion path (Fig. 1G), suggesting possible fiber loss and interruption of neural projections in the ventricular parenchyma. These findings suggest abnormal development of the thalamus/diencephalon in *c-Jun* KO mouse embryos, accompanied by nerve fiber loss in adjacent parenchyma.

**Fig. 2:**
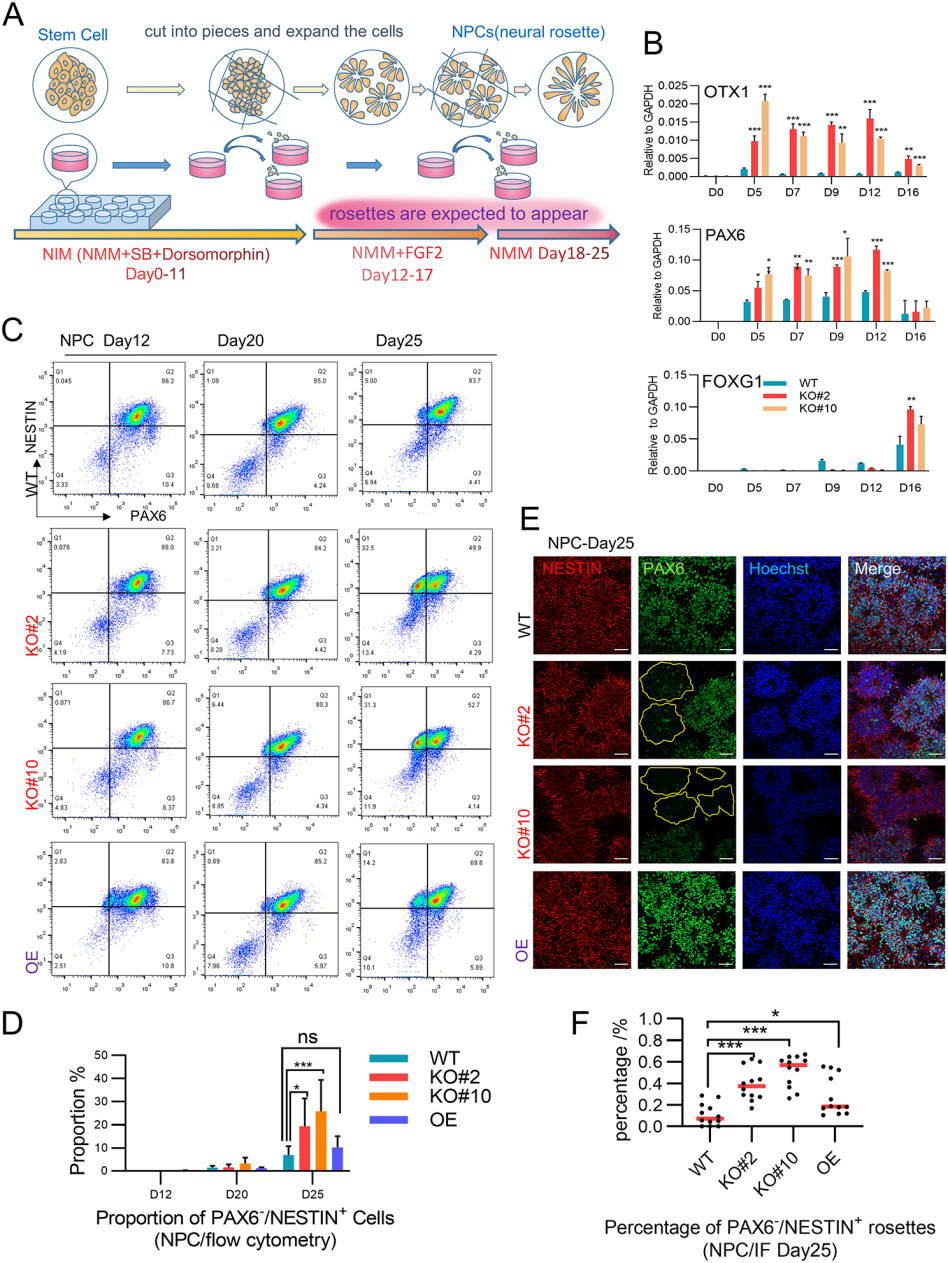
Loss of *c-JUN* in NPCs promoted neural stem cell induction and accelerated PAX6^+^cell differentiation. (**A**) Schematic of NPC induction. NIM: neural induction medium, NMM: neural maintain medium, SB: SB431542 (**B**) RT-qPCR gene expression test revealed that several NPC marker genes were significantly upregulated in the KO groups. (3 independent experiments). The values are presented as the mean ± SD. *p < 0.05, **p < 0.01, ***p < 0.001. (**C**) Flow cytometry plots illustrate the distribution of cells stained with PAX6 and NESTIN on day 12, 20, and 25. Differentiated PAX6^−^/NESTIN^+^ subcluster appeared in the KO groups, but were partially rescued in the OE groups on day 25. (**D**) Statistical analysis of the flow cytometry data showed a significant increase in cells within Q1 quadrant (PAX6^−^/NESTIN^+^ cells) (3 independent experiments). Two-way ANOVA. The values are presented as the mean ± SD, *p < 0.05, **p < 0.01, ***p < 0.001. (**E**) IF staining showed an increase in PAX6^−^/NESTIN^+^ rosettes (circled with yellow lines) in the KO groups on day 25. Scale bar: 50 μm. (**F**) Statistical analysis of IF staining showed a significant increase in PAX6^−^/NESTIN^+^ rosettes in the KO groups, which is partially reversed in the OE groups on D25 (*n* = 12, 4 independent experiments). *p < 0.05, **p < 0.01, ***p < 0.001

**Fig. 3:**
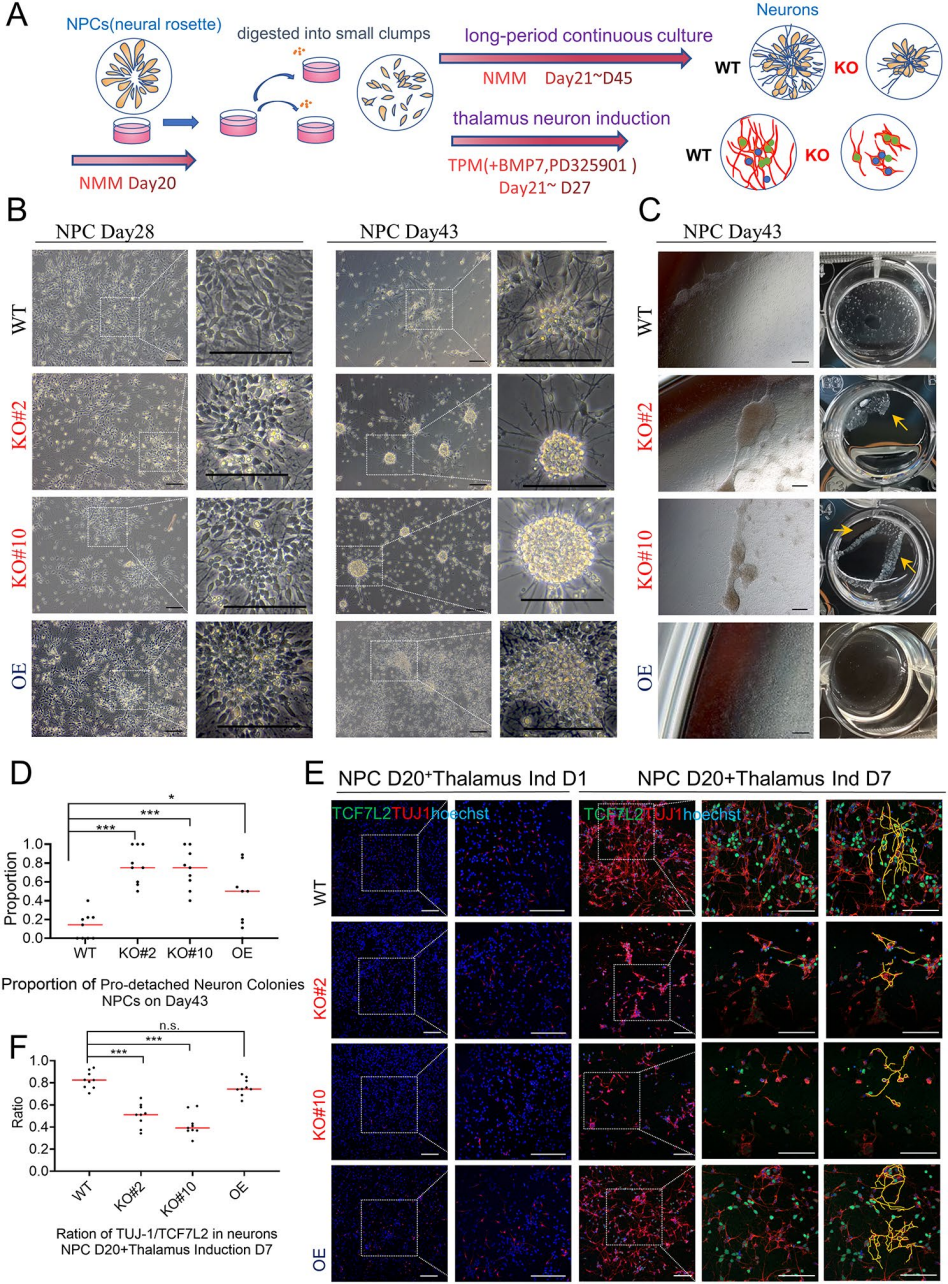
Loss of *c-JUN* weakened the nerve fiber extension and adherent ability on plates in the neural culture. (**A**) Schematic of long-period continuous culture and thalamus neuron induction. NMM: neural maintain medium, TPM: thalamus patterning medium. (**B**) BF view of NPCs on day 28 (left panel) and day 43 (right panel). NPCs in the WT, KO, and OE groups could spread flat on the plates and extended plentiful fibrous axons on D28. In the KO groups, the cell bodies were more likely to aggregate and the axonal fibrous were significantly shrunk on day 43. Scale bar: 100 μm. (**C**) BF view of curled-up edges of cell colonies in the KO groups but not in the WT or OE group. Cells in the KO groups were easily detached from the culture dish (12-well dish) when subjected to enzymatic digestion. The white film indicated by the arrowheads shows cells shed after digestion. (**D**) Statistical analysis of the proportion of pro-detached colonies in NPCs on day 43. The KO groups showed a significantly high proportion of pro-detached colonies (3 independent experiments, 9 fields of view per group). *p < 0.05, **p < 0.01, ***p < 0.001. (**E**) IF staining of TUJ1 and TCF7L2 neurons on thalamus induction D1 and D7, respectively. In order to clearly mark the distribution of nerve fibers, TUJ1^+^ signals are highlighted by yellow lines (the rightmost column) Scale bar: 100 μm. (**F**) Statistical analysis of the ratio of the signal area of TUJ1 to TCF7L2 in NPCs on thalamus induction D7. The KO groups showed a significantly reduced ratio. This pattern was restored in the OE group. (3 independent experiments, 9 fields of view per group). *p < 0.05, **p < 0.01, ***p < 0.001, n.s.: not significant. BF, bright-light field

The temporal and spatial expression of *c-Jun*, *Pax6* (a key gene in NPCs/neuron development), and *Tcf7l2* (a thalamus-associated neural gene) was analyzed using the Mouse Organogenesis Spatiotemporal Transcriptomic Atlas (MOSTA) [21]. The data revealed the spatiotemporal expression patterns of these genes in mouse embryonic development from E9.5 to E16.5. IF staining on mouse brains at E14.5 confirmed their expression patterns as described by MOSTA (Fig. S2A, B). Our findings show that *c-Jun* is widely expressed in the embryonic development, with a stronger distribution in the subventricular zone in the forebrain cortex adjacent to diencephalon after E14.5. *Pax6* expression is primarily detected in the ventricular lateral cortex of the forebrain and part of diencephalon, while *Tcf7l2* is mainly found in the thalamic region of the diencephalon. Overlap in the expression of *c-Jun* and *TCF7L2* was observed in the early developmental stage (E11.5 ~ 12.5) (Fig. S2C, D). The *c-Jun* KO may interfere with this relationship, contributing to the developmental abnormalities observed in the thalamus/dicephalus region.

### Loss of *c-JUN* weakened the nerve fiber’s extension

To investigate the role of *c-JUN* in human neural development, we generated *c-JUN* KO stem cell clones (KO#2, KO#10) from H1 ESCs using the CRISPR-Cas9 system, as previously described [12]. The karyotypes and cell proliferation/self-renewal of these KO clones appeared normal compared to WT H1 ESCs (Fig. S3A). Confirmation of *c-JUN* KO at the protein level was achieved through western blot analysis (Fig. S3B). Null expression of *c-JUN* mRNA in KO groups was verified by quantitative reverse transcription-polymerase chain reaction (RT-qPCR), whereas WT groups exhibited an upward trend of *c-JUN* expression throughout neural induction (Fig. S3C). During NPC induction (Fig. 2A), NESTIN and PAX6 signals were detected in both WT and KO H1 ESCs as early as day 4 with no significant differences. By around day 12, round, clustered neural rosettes began to appear, and by day 17, neural rosettes with a radial pattern expressing neural stem cell markers NESTIN and PAX6 were uniformly spread (Fig. S3E). After dispersing and replanting, typical cluster structures of rosettes were observed by day 23 (Fig. S3F). Notably, at the early stage (< day 20), complete depletion of *c-JUN* did not impede neural fate determination, and NPCs could generate typical PAX6^+^/NESTIN^+^ neural rosettes. Although rosette morphology showed no discernible differences before day 20, KO groups exhibited significantly increased expression of *OTX1*, *PAX6*, and *FOXG1* tested by RT-qPCR (Fig. 2B). As the culture progressed, flow cytometry analysis at day 12, day 20, and day 25 identified a new sub-cluster appearing in the Q1 region in KO cells, indicating PAX6^−^/NESTIN^+^ cells (Fig. 2C), with the proportion of these cells significantly higher in KO than in WT on day 25 (Fig. 2D). This new sub-cluster was confirmed by IF staining, with some PAX6^−^/NESTIN^+^ rosettes observed in KO groups on day 25. These rosettes still exhibited NESTIN^+^ outlines, whereas internal PAX6 signals were significantly weakened or disappeared (Fig. 2E). Although PAX6^−^/NESTIN^+^ NPCs could occasionally be detected in the WT group, their proportion was much lower (Fig. 2F). Moreover, we established a tetracycline-inducible system to overexpress (OE) *c-JUN* using doxycycline (DOX). Western blot analysis confirmed the OE of *c-JUN* protein in KO cell lines (Fig. S3D). Upon OE of *c-JUN* in KO groups, the proportion of PAX6^-^/NESTIN ^+^ cells reduced to a level comparable to the WT group, and the percentage of PAX^-^/NESTIN ^+^ rosettes was also reduced to a level close to the WT group. These data indicate that the pro-differentiated NPCs were partially rescued (Fig. 2C-F). These findings suggest that c*-JUN* KO accelerates the induction of NPCs, and mature neural stem cells may be more prone to further differentiation.

When NPCs were cultured continuously (Fig. 3A), both WT and KO cells grew axons with typical neuronal morphology in the early days (D26–D35) (Fig. 3B, left panel). However, after day 35 without renewing the plate, WT cell axons continued to spread on the culture dish and extended plentiful fibrous axons that interwove with each other (Fig. 3B, right panel). In contrast, in KO groups, the axonal weaves significantly shrunk, and neuronal cell bodies tended to aggregate, with more cell colonies showing a tendency to curl up (Fig. 3B, right panel). We defined the neuronal colonies that were obviously aggregated with smooth edges and had a tendency to detach as “pro-detached colonies.” Statistical analysis of the proportion of pro-detached colonies in random field views revealed a significantly higher proportion in the KO groups (Fig. 3D). Moreover, when cells were subjected to enzymatic digestion (Accutase, STEM CELL, 07920) for 5 min at 37 °C, cells in KO groups detached more easily from the culture dish (Fig. 3C). This pro-aggregated and easier-detached phenomenon observed in long-period culture was rescued in OE groups (Fig. 3B-D). It is worth mentioning that neurons in all groups eventually aggregated and fell off, likely due to the exhaustion of coating proteins (e.g., laminin) in long-time cultures without changing plates. However, this process occurred significantly earlier in the KO groups. Furthermore, NPCs were induced into TCF7L2^+^ thalamus neurons using thalamus patterning medium (TPM) as used in ThO induction (Dulbecco’s Modified Eagle Medium [DMEM]/F12 medium containing N2, B27 without vitamin A, BMP7, PD325901, etc.) (Fig. 3A). Initially (day 21), newly seeded NPCs demonstrated undetectable TCF7L2 signals on cell bodies and weak class III beta-tubulin (TUJ1) signals along with short fibers (Fig. 3E, left panel). Following 7 days of induction, some cells expressed TCF7L2 signals along with abundant TUJ1^+^ nerve fibers (Fig. 3E, right panel). The ratio of TUJ1 signals to TCF7L2 signals was significantly lower in KO groups (Fig. 3F). This suggests that the TUJ1^+^ membrane and fibers (red signals) around the same number of TCF7L2^+^ neuron bodies (green signals) were significantly reduced in KO neurons. In 2D thalamus neuron differentiation, *c-JUN* KO restrained the initiation and extension of nerve fibers. Continuous culture of these TCF7L2^+^ thalamus neurons without renewing the plates in neural differentiation medium (the medium used in ThO induction in later stages) also revealed more aggregation and earlier shedding colonies in KO groups at day 43, similar to the phenomenon observed in long-period continuous NPCs culture. These findings collectively indicate that absence of *c-JUN* impairs nerve fiber extension and weakens neurons’ adhesive capabilities to the substrate, resulting in a propensity for neuronal colonies to aggregate and detach more readily from the culture plate.

### COs derived from *c-JUN* KO hESCs exhibited robust exteriors but loose interiors

To assess the impact of *c-JUN* on neural development within a model that closely mimics physiological conditions, we generated COs in an unguided manner (Fig. S4A). This whole-brain model exhibits preliminary ventral and dorsal axes and regional aggregation phenomena, with neural connections between the regions [22–24]. IF staining revealed the distribution of c-JUN in WT CO sections on day 12, 30 and 50 (Fig. S4B) and RT-qPCR test confirmed *c-Jun* expression increased during CO induction, while it was absent in the KO groups (Fig. S4C). In COs derived from H1ESCs, IF analysis detected neural stem cells markers (NESTIN, PAX6, SOX2, phospho-vimentin), neuronal marker (TUJ1), astrocytic marker (GFAP), and forebrain or cerebral cortex markers (FOXG1, TBR1, TBR2) within radially enlarged cavities and layered structures on day 30 and day 50. This suggests that the cultured COs exhibited cellular composition and morphogenetic patterns resembling early brain development (Fig. S4D-F) [24, 25]. Downregulation of stem cell-enriched genes (*OCT4* and *CK8*) by day 16 and upregulation of nerve-related genes (*PLAGL1* and *TBR-2*) from day 16 to day 30 were confirmed by RT-qPCR (Fig. S4G). Furthermore, at later stages, marker related to the forebrain cortex and synaptic function, such as *TBR-1*, *CTIP-2*, and *SYNAP* (D30–D60), were detected from day 30 to day 60 (Fig. S4H). These findings outlined a initial in vitro model of early cerebral development (Fig. S4I).

**Fig. 4:**
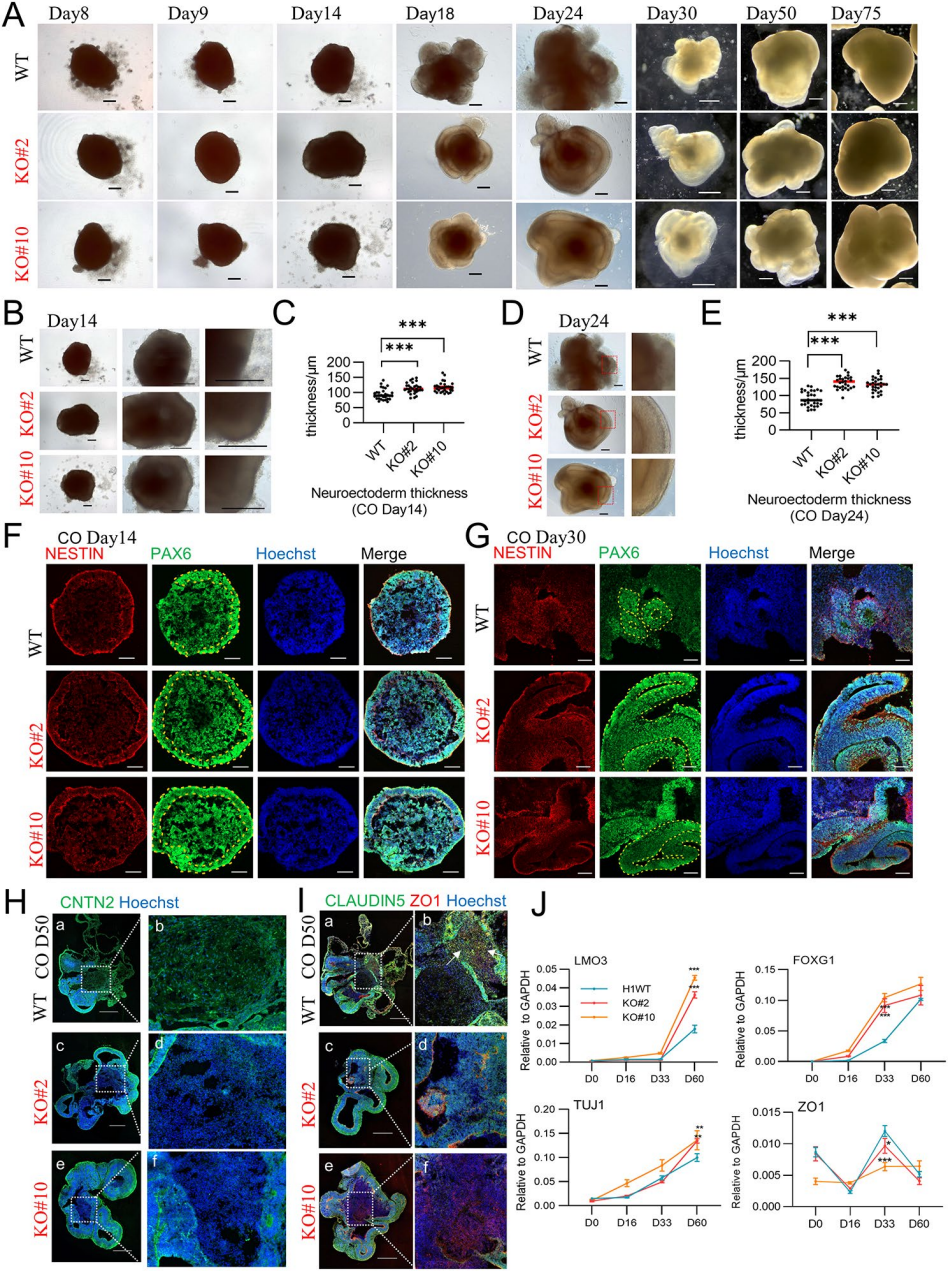
KO-derived CO showed a robust exterior, but less fiber-connected core. (**A**) Bright field view of CO induction from WT and KO cell lines., Scale bar: 200 μm for day 8–24; Scale bar: 500 μm for day 30–75. (**B**–**C**) KO groups showed a significant increase in the width of the neural ectoderm at day14 (*n* = 30, 3 independent experiments). *p < 0.05, **p < 0.01, ***p < 0.001. (**D**, **E**) The KO groups showed a significantly increased width of the expanded neural cortex-like structures at day30 (*n* = 30, 3 independent experiments). *p < 0.05, **p < 0.01, ***p < 0.001. (**F**, **G**) IF staining of CO on D14 and D30. The KO groups show robust PAX6^+^/NESTIN^+^ closed-ring shape ectoderm and increased width of PAX6^+^/NESTIN^+^ neural cortex-like layer (circled with yellow dashed line in the PAX6 channel). Scale bar: 100 μm. (**H**) IF staining of CNTN2 in COs at day 50. In WT, CNTN2 could be detected both in the periphery region and the interior (a, b). In the KO groups, CNTN2 was most distributed in the periphery but not in the core (c–f). Scale bar: 500 μm. (**I**) IF staining of ZO-1, CLAUDIN5 in COs at day 50. WT group showed tight-junction rich signals in the outer neural cortex-like layer as well as in the interior (indicated by the white arrows in b). In the KO groups, the signals were detected mostly on the outer layer, but not in the core. Scale bar: 500 μm. (**J**) qRT-PCR gene expression test revealed that several NPC/neuron marker genes (*LMO3*, *FOXG1*, and *TUJ1*) were upregulated while tight junction genes (ZO-1) were downregulated in the KO groups during CO induction (3 independent experiments). The values are presented as the mean ± SD. *p < 0.05, **p < 0.01, ***p < 0.001

When comparing COs derived from WT and *c-JUN* KO cells, a more prominent transparent ring-shaped outer layer was observed in the KO on day 12–14 during CO culture (Fig. 4A-C). By day 24, an expanded transparent fan-shaped structure appeared in the KO, which was significantly wider than that in the WT group (Fig. 4D, E). IF analysis confirmed these observations during the corresponding period, showing that the transparent outer layer on day 12–14 was composed of PAX6^+^/NESTIN^+^ neural ectoderm (Fig. 4F). In COs derived from *c-JUN* KO cells, the neural ectoderm formed a robust closed ring on the outermost layer, whereas this ring was less robust and commonly interrupted in WT-derived COs (Fig. 4F). Subsequent structures derived from this ring in KO samples exhibited a significantly increased width of the PAX6^+^/NESTIN^+^ layer and enlarged cavities on day 30 (Fig. 4G). We further examined several neuronal and structural protein markers. Contactin protein 2 (CNTN2), which is predominantly expressed in the brain and encodes a glycosylphosphatidylinositol-anchored neuronal membrane protein involved in the proliferation, migration, and axon guidance of neurons during cerebral development [26, 27], was distributed in both the outer layer and some parts of the inner core in WT samples (Fig. 4H, a, b). However, in KO-derived COs, CNTN2 was primarily distributed in the outer cortical-like spheres (Fig. 4H, c-f). Similar distribution patterns were observed for the protein markers CLAUDIN5 and ZO1. In WT samples, CLAUDIN5 and ZO1 signals were detected in the outer layer as well as in the low-cell-density inner core (Fig. 4I, a, b, white arrows indicated). Conversely, in KO-derived COs, the signals were predominantly distributed in the cortex-like structures (Fig. 4I, c-f). Overall, RT-qPCR revealed the upregulation of neural marker genes such as *LMO3*, *FOXG1*, and *TUJ1*, whereas tight junction-related genes such as *ZO-1* were downregulated in KO during CO induction (Fig. 4J).

These results indicate that COs derived from *c-JUN* KO cells exhibit a more robust neural ectoderm and extended subsequent neural structures but possess a less fibrous connected core. This trend aligns with the observations in *c-JUN* KO mouse embryos, where the cortex remained unchanged, whereas the ventricular parenchyma, particularly structures adjacent to the thalamus in the diencephalon, exhibited increased sparsity.

### *c-JUN* KO upregulates nervous system development-related pathways and downregulates cell–cell adhesion pathways

**Fig. 5:**
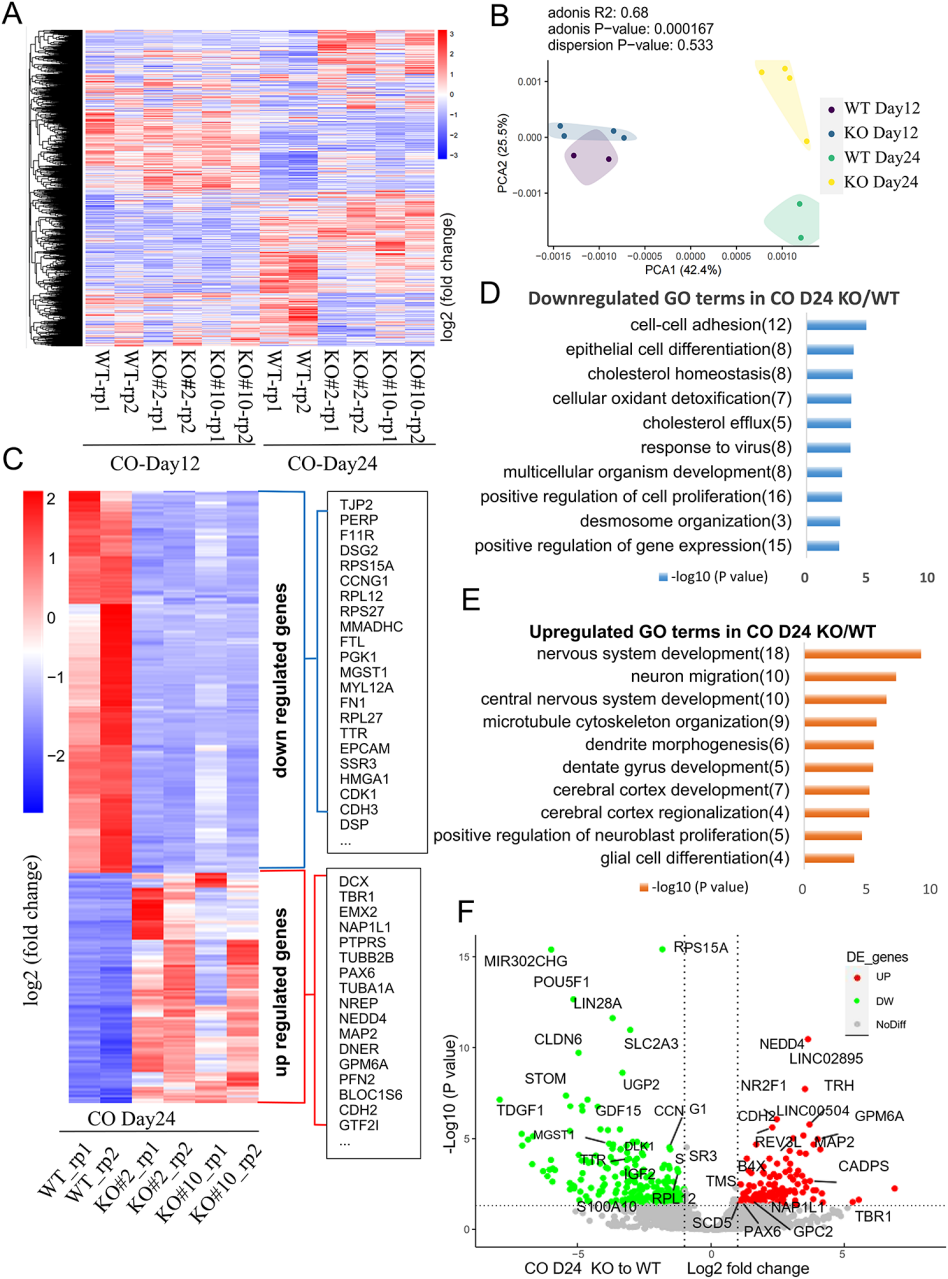
*c-JUN* KO upregulated the pathways of nervous system development but downregulated the pathways in cell-cell adhesion. (**A**) Heat map of CO transcriptome sequencing on D12 and D24. (**B**) PCA analysis revealed the principal components clustered separately according to the two time points, and the divergence was obvious on D24 between the WT and KO groups. (**C**) Heat map of differentially expressed genes (DEG) in CO D24. (**D**, **E**) GO-BP analysis of DEG in CO D24. When compared to WT, the top 10 downregulated and top 10 upregulated biological pathways in KO are listed respectively. (**F**) Volcano map of DEG in CO day24

To further understand the role of *c-JUN* in neural development, we conducted RNA-seq analysis on both WT and KO-derived COs at day 12 and day 24. Gene expression analysis using heat maps and principal component analysis (PCA) revealed significant differences between WT and KO samples at day 24 (Fig. 5A, B). Differentially expressed gene (DEG) analysis identified 141 genes, including *MAP2*, *PAX6*, *DCX*, and *TBR1*, that were significantly upregulated (*p* < 0.05, Fold change [FC] ≥ 2) in KO-derived COs compared to WT (Fig. 5C). Notably, other *c-JUN* family members *JUNB*, *JUND*, *c-FOS*, and *FOSB* exhibited no significant changes (data not shown). Gene Ontology (GO) analysis indicated that the upregulated genes in KO-derived COs were enriched in terms related to nervous system development, neuron migration, CNS development, and cerebral cortex development (Fig. 5E). This included genes involved in cerebral cortex development such as *DCX*, *TBR1*, and *EMX2*. Conversely, 223 genes, including *RPS15A*, *CCNG1*, *FTL*, *TTR*, and *CDK1*, were significantly downregulated (*p* < 0.05, FC ≥ 2) in KO-derived COs and were enriched in GO terms related to cell–cell adhesion, epithelial cell differentiation, and cholesterol homeostasis (Fig. 5C, D). The highest-ranking group, cell–cell adhesion, included genes such as *TJP2* (also known as *ZO-2*), *PERP*, *F11R*, and *DSG2*, which play critical roles in maintaining tight cell connections. The volcano plot of DEGs in COs at D24 illustrated their distribution pattern and highlighted several genes (Fig. 5F). The up-regulated genes including *NEDD4*, NEDD4 E3 ubiquitin protein ligase, play critical roles in the regulation of a number of membrane receptors, endocytic machinery components and the tumor suppressor PTEN [28, 29]. *TRH*, thyrotropin releasing hormone, encodes a member of the thyrotropin-releasing hormone family. Thyrotropin-releasing hormone is involved in the regulation and release of thyroid-stimulating hormone, as well as prolactin. Deficiency of this hormone is associated with hypothalamic hypothyroidism [30, 31]. *MAP2*, microtubule associated protein 2, belongs to the microtubule-associated gene family and is thought to be involved in microtubule assembly, playing essential role in neurogenesis [32]. The down-regulated genes including *POU5F1*, POU class 5 homeobox 1, also known as *OCT4*, encodes a transcription factor containing a POU homeodomain that plays a key role in embryonic development and stem cell pluripotency. Aberrant expression of this gene in adult tissues is associated with tumorigenesis [33, 34]. *CLDN6*, claudin 6, a tight junctions protein, represents one mode of cell-to-cell adhesion in epithelial or endothelial cell sheets, forming continuous seals around cells and serving as a physical barrier to prevent solutes and water from passing freely through the paracellular space [35].

Previous studies have reported that *c-JUN* suppresses epithelialization [11, 36]. However, our GO analysis of COs at day 24 showed a group of epithelial marker genes were down-regulated (Fig. 5D). The neuroepithelium may be quite different from the general epithelialization or mesenchymal-to-epithelial transition. Factors such as cell types, differentiation stage, and cellular microenvironment can vary in gene functions and outcomes. We examined those 8 genes catalogued as “epithelial cell differentiation” in terms of significance ranking (*p* value), from high to low: *F11R*, *LGALS3*, *KRT19*, *GSTA2*, *PGK1*, *CDK1*, *GSTA1*, *ELF3*. Substantial evidence suggests they also play strong roles in structure maintenance and cells connection [37–42]. Thus, we hypothesize that in our neural developmental model, the down-regulated genes due to KO are likely associated with structural/cell adhesion pathways, and may differ from general epithelial differentiation and formation.

Overall, these data suggest that *c-JUN* functions as a regulatory hub closely associated with neurodevelopmental processes and the maintenance of cellular structure. *c-JUN* KO resulted in the upregulation of neurodevelopment-related pathways while downregulating cell–cell adhesion pathways in a model of cerebral development.

### *c-JUN* KO affected the TCF7L2^+^ cell distribution in COs and mouse embryos

**Fig. 6:**
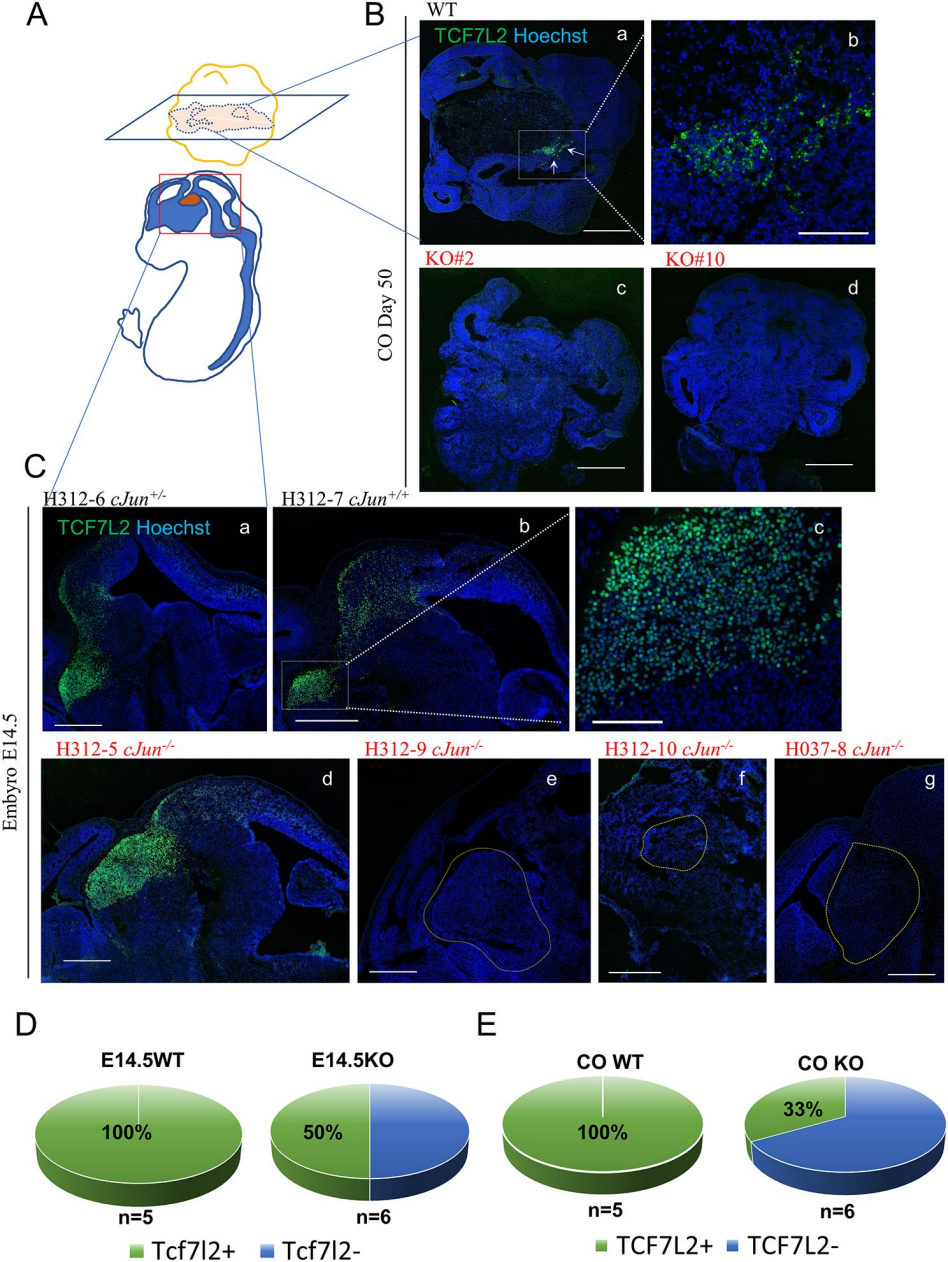
The disturbed expression pattern of thalamus/diencephalon marker TCF7L2 in*c-Jun* KO embryos and CO. (**A**) A diagram of the CO section and mouse embryo section; the red color area in the embryo section shows the thalamus in diencephalon. (**B**) WT-derived CO D50 could detect TCF7L2 signals, implying primary diencephanlic cells near the central area (a, b), while TCF7L2 was hardly detected in the KO groups. Image b is a partially enlarged image of the white arrow-indicated area in the image (a) Scale bar: 500 μm for a, c, d; 200 μm for (b) (**C**) WT embryos at E14.5 could detect TCF7L2 signals, implying thalamus in the diencephalon (a, b, c), while KO embryos appeared different in the staining cases: H213-5 showed TCF7L2 signals in the diencephanlic area (d); H312-9 and H312-10 have negligibly detected TCF7L2 signals (e, f). Another KO embryo E14.5 H037-8 from another litter showed undetectable TCF7L2 signals in the corresponding area (g). Image c is a partially enlarged image of image b. Scale bar: 500 μm for a, b, d–g; Scale bar: 100 μm for (c) (**D**) Statistical analysis of the proportion of TCF7L2^+^ cells detectable samples in embryos at E14.5. (**E**) Statistical analysis of the proportion of TCF7L2^+^ cells in COs on day 50

*TCF7L2* is known to be crucial for the development of forebrain and thalamus [43, 44], and it is commonly used as a marker specific for thalamus [45]. In embryos at E14.5 from the same litter H312, both WT embryos H312-7 (*c-Jun*^*+/+*^) and embryos H312-6 (*c-Jun*^*+/−*^) exhibited detectable TCF7L2^+^ signals in the ventral diencephalon (Fig. 6C, a, b, c). However, in the KO embryos, different staining patterns were observed: H213-5 (*c-Jun*^*−/−*^) displayed TCF7L2^+^ signals in the diencephalic area (Fig. 6C, d), whereas H312-9 (*c-Jun*^*−/−*^) and H312-10 (*c-Jun*^*−/−*^) showed hardly any detectable TCF7L2 signals in the expected areas (Fig. 6C, e, f). The TCF7L2 signals in another embryo at E14.5, H037-8 (*c-Jun*^*−/−*^), from a different litter, were also undetectable in the corresponding area (Fig. 6C, g). This discrepancy was partially mirrored in CO models: TCF7L2^+^ signals were present in WT-derived COs at day 50, particularly in subcortex-like regions and the outer margin of the core, reminiscent of the brain ventricular parenchyma, such as the diencephalon and thalamus (Fig. 6B, a, b). Conversely, TCF7L2 signals were scarcely detected in KO-derived COs (Fig. 6B, c, d). Analysis of 11 mouse embryos and 11 CO samples revealed that TCF7L2^+^ cells were detected in all WT samples, whereas clearly identifiable TCF7L2 signals were observed in only 50% of KO mouse embryos and 33% of KO CO samples (Fig. 6D, E). These findings indicate that abnormalities in the thalamus of the diencephalon may be traced back to aberrantly developed and distributed TCF7L2^+^ cells at the cellular level. The consistency between the trends observed in mouse embryos and COs suggests that CO models partially replicate differences observed in vitro.

### *c-JUN* KO hindered thalamus development in ThOs

**Fig. 7:**
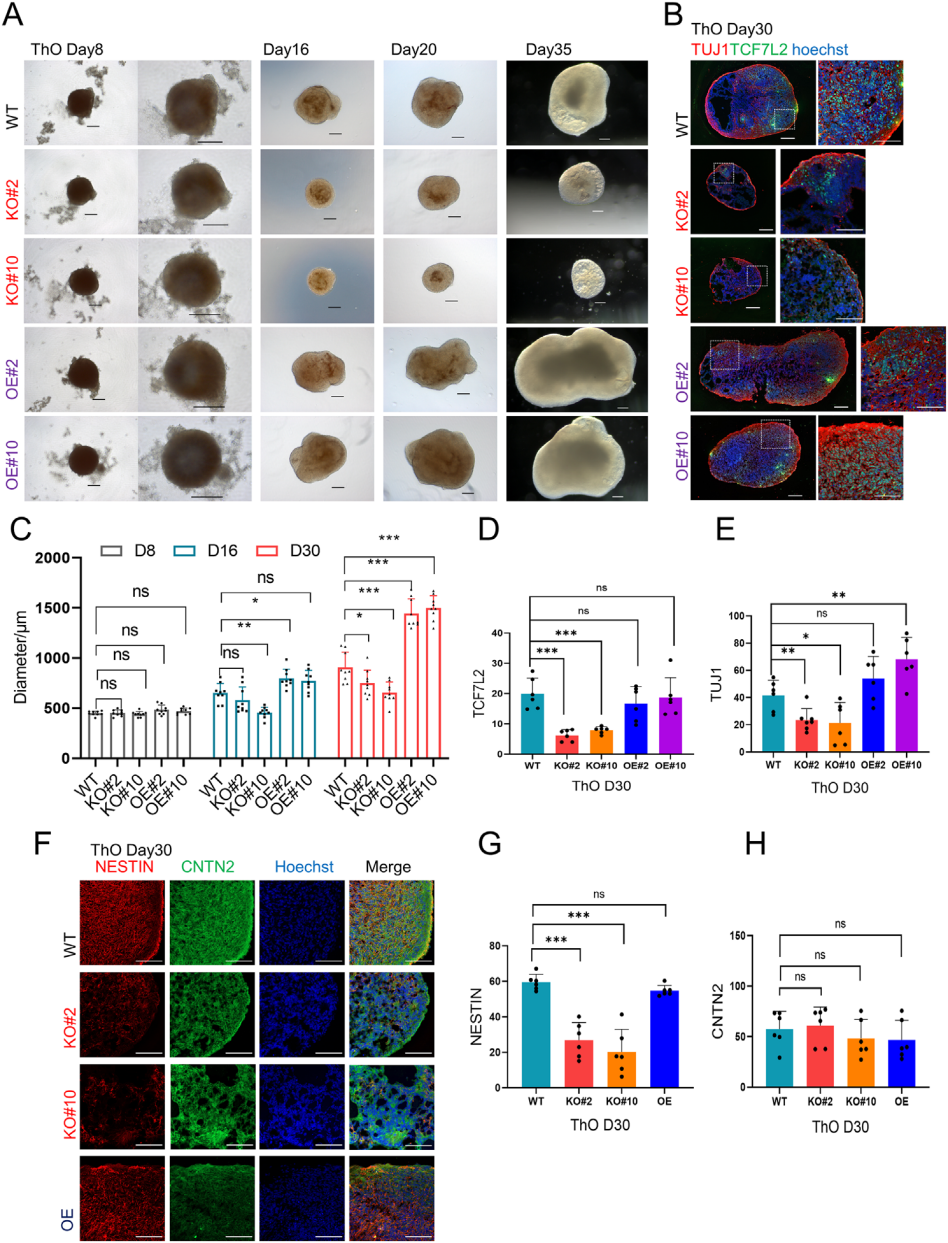
*c-JUN* KO-derived ThO showed a hindered sphere in diameter and sparse internal fibers. (**A**) Global overview in the bright field of ThO induction from WT, KO, and OE cell lines. day 8–35, Scale bar: 200 μm. (**B**) IF staining of ThO revealed robust TUJ1^+^/TCF7L2^+^ cells in the WT and OE groups on D30, while the KO groups showed a decreased density. Scale bar: 100 μm. (**C**) Statistics analysis of the diameter of ThO in WT, KO, and OE under a bright-field view on days 8, 16, and 30. The KO groups showed a significantly smaller diameter (*n* = 9, 3 independent experiments). (**D**, **E**) Statistics analysis of IF staining of TCF7L2 and TUJ1 in ThOs in WT, KO, and OE on day 30. The KO group showed a significantly reduced TCF7L2 and TUJ1 signals (*n* = 6, 3 independent experiments). (**F**) IF staining of NESTIN and CNTN2 in ThO on day 30. Scale bar: 100 μm. (**G**, **H**) Statistics analysis of IF staining of NESTIN and CNTN2 in ThOs in WT, KO, and OE on day 30. The NESTIN signal in KO groups was decreased, whereas there was no significant difference in the CNTN2^+^ signal among three groups. Scale bar: 100 μm. (*n* = 6, 3 independent experiments). Values are presented as the mean ± SD. *p < 0.05, **p < 0.01, ***p < 0.001

The evidence from mouse and CO models suggests that the loss of *c-JUN* function influences the development of the diencephalic thalamus. To investigate the direct differentiation of ThO, we referred to recently published organoid protocols and established a guided ThO model [46, 47]. A schematic and overall view of ThO induction from H1 ESCs at D8–D35 are depicted (Fig. 7A, Fig. S5A). IF staining of ThOs on day 20 revealed TCF7L2^+^ cells in both the upper (Fig. S5B, a) and internal spheres (Fig. S5B, b). IF staining on day 30 detected the presence of c-JUN, ZO1, Claudin5, Collagen type-IV, TUJ1, NESTIN, and CNTN2 in H1 ESC-derived ThOs (Fig. S5C, D, E). An overall view under bright field illumination of Matrigel-embedded COs and ThOs from H1 ESCs on day 26 (co-culture day 4) showed that ThOs, but not COs, could form fibra-like structures (Fig. S5F, a, b), consistent with the role of the thalamus in transmitting signals via thalamocortical axon (TCA) projections, serving as a CNS relay system. These tests validated the successful establishment of human ThOs.

**Fig. 8:**
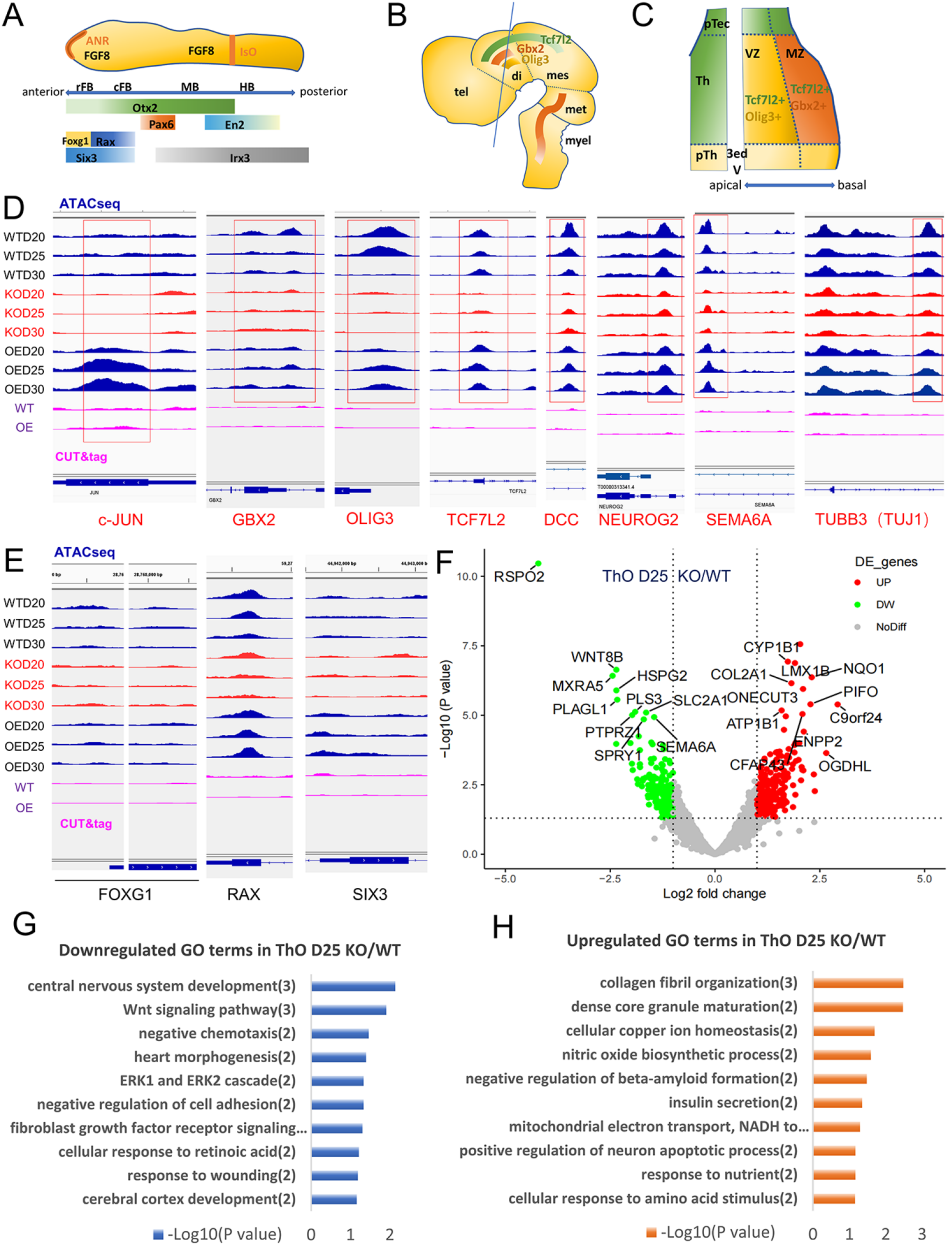
ATAC-seq revealed that *c-JUN* KO restrained the thalamus gene expression in ThO. (**A**) Schematic of the characteristic signal distribution in the telencephalon and diencephalon regions in the early embryonic neural tube. ANR, anterior neural ridge; IsO, isthmic organizer; rFB, rostral forebrain; cFB, caudal forebrain; MB, midbrain; HB, hindbrain; (**B**, **C**) Schematic representation of the characteristic gene expression patterns from a lateral view (**B**) and the frontal section (**C**). The solid line in B represents the plane of the frontal section in **C**. tel, telencephalon; di, diencephalon; mes, mesencephalon; met, metencephalon; myel, myelencephalon; 3rd V, third ventricle; mz, mantle zone; vz, ventricular zone; pTec, pretectum; pTh, prethalamus; Th, thalamus; (**D**, **E**) ATAC-seq revealed that chromatin accessibility was reduced in genes related to thalamus development and axon projections in the KO groups and restored after over-expression but no difference (*FOXG1*, *SIX3*) or slight reduction (*RAX*) in the genes relate to telencephalon development. Lanes 1–9 (blue or red) were ATAC-seq data and lanes 10–11 (pink) were CUT&Tag data. (**F**) Volcano plot of DEG from RNAseq in ThOs D25. Multiple genes were significantly differentially expressed in the KO group in thalamus organoid development on D25. (**G**, **H**) GO analysis of RNA-seq data obtained from ThO D25, *c-JUN* KO to WT. fibroblast growth factor…: fibroblast growth factor receptor signaling pathway (2); mitochondrial electron…: mitochondrial electron transport, NADH to ubiquinone (2)

When initiating differentiation using WT and *c-JUN* KO cell lines, all cell lines formed similar embryoid bodies (EBs) initially. However, the development of organoids in the KO groups was significantly hindered when entering the thalamus patterning stage, which is pivotal for thalamic cell fate determination (Fig. 7A, C). By day 16, the diameter of organoids in the KO#10 group was significantly smaller than that in the WT group. By day 30, the diameter of organoids in both KO groups was markedly smaller than that in the WT group (Fig. 7A, C). This growth lag was reversed upon *c-JUN* OE, and the organoid diameter in the OE groups even surpassed that in the WT group (Fig. 7A, C). Regarding the internal cell organization, the cell density in the KO group was notably reduced, as evidenced by diminished expression of neural-specific markers such as TCF7L2 and TUJ1 (Fig. 7B, D, E), or nerve fiber marker NESTIN, whereas the CNTN2^+^ signals showed no significant differences. (Fig. 7F-H). This defect was rescued in the *c-JUN* OE group (Fig. 7C, E). This phenotype suggests that *c-JUN* deletion impedes the formation and development of thalamus tissue, which is consistent with the observations in mice and COs.

*c-JUN* serves as a crucial transcription regulator by binding to DNA through leucine zipper-forming dimers [48, 49]. Thus, it is imperative to analyze the on/off status and c-JUN binding status of relevant genes during differentiation from the perspective of genome accessibility. To achieve this, we conducted ATAC-seq, a method for mapping chromatin accessibility genome-wide [50, 51]. Our analysis of ThO at day 25 during development revealed several significant findings. First, ATAC-seq confirmed the high chromatin accessibility of *c-JUN* in WT and OE groups, whereas less accessibility in KO groups (Fig. 8D). This indicated the direct involvement of *c-JUN* in ThO induction. In terms of thalamus development regulation, according to a concept maps showing several characteristic signals distribution in the telencephalon and diencephalon regions during early CNS development (Fig. 8A-C) [52], the chromatin accessibility of key genes including *GBX2*, *OLIG3*, and *TCF7L2* was remarkable reduced in ThOs in the KO groups compared to WT groups and restored in OE groups (Fig. 8D). This reduction suggests that the expression of key genes in the thalamus and its precursors was downregulated due to *c-JUN* KO, which may account for the suppressed volume of ThOs observed under bright-field microscopy. Moreover, genes related to axon growth and TCA projection such as *DCC*, *NEUROG2*, *SEMA6*, and *TUBB3* [53–55] were also reduced in KO groups and restored after OE (Fig. 8D). This is in line with the sparse nerve fibers observed in KO ThOs in IF staining. In terms of telencephalon-related genes, there were no significant changes in *FOXG1* and *SIX3*, and a slight reduction in *RAX* was detected (Fig. 8E), suggesting that the telencephalon or forebrain may not be affected, which was partially consistent with the observed phenotype in mice and COs. We also performed a CUT&Tag assay aiming to detect the binding status of c-JUN protein on the genome [56] during ThO development. Preliminary results showed weak signals at gene loci including *TUBB3*, *DCC*, *SEMA6A*, and *SIX6* (Fig. 8D, E), indicating potential direct binding of c-JUN protein to these genes. Additionally, RNA-seq analysis identified significant differential expression of multiple genes in the KO ThO at day 25 compared to the WT group. This included downregulation of genes such as *PSRO2*, *WNT8B*, *MXRA5*, and *PLAGL1*, and upregulation of genes such as *MSX1*, *CYP1B1*, *LMX1B*, and *COL2A1* (*p* < 0.05, FC ≥ 2) (Fig. 8F). The volcano plot depicted their distribution patterns (Fig. 8F). GO analysis listed the top 10 downregulated and upregulated biological pathway terms, respectively (Fig. 8G, H). In summary, our comprehensive analysis using ATAC-seq, CUT&Tag assay, and RNA-seq confirmed that *c-JUN* KO affects the expression of multiple genes at both the chromatin and mRNA levels during thalamus induction, and the disruption of this regulation may contribute to the hindered development of ThO.

These findings, in conjunction with consistent effects observed in 2D cells, 3D organoids, and animal models, underscore the critical role of *c-JUN* in early neural development. They suggest that the loss of *c-JUN* function may impede thalamus development in human organoids and mouse models.

## Discussion

From the comprehensive analysis of multiple models, our study has highlighted the significant impact of *c-Jun* loss on brain development, particularly in the diencephalic thalamus. The consistent trends observed across various neurodevelopmental models suggest that thalamus development is particularly susceptible to *c-Jun* deficiency, potentially leading to disruptions in both development and the maintenance of established structures. The observed attenuation of axonal growth and compromised structural maintenance likely underlie these effects.

Previous studies have reported embryonic lethality in *c-Jun* KO mice during the second week of embryonic development, with no significant differences reported in the central nervous region [18, 19]. However, the larger intercellular spaces observed in KO brains were once attributed to edema [19]. More recent studies using conditional KO models based on the c-Jun^f/f^-cre system have demonstrated normal global brain histology and expression of neural markers such as Neuronal nuclear antigen (NeuN), MAP2, and GFAP, except for increased facial motoneurons [57].

Further research has highlighted the high expression of *c-Jun* in response to neuronal trauma, suggesting its role in axonal regeneration [58]. In *c-Jun* deficient motor neurons, atrophy and reduced muscle reinnervation were observed [58]. *c-Jun* N-terminal kinase (JNK), a regulator of *c-Jun*, binds to the *c-Jun* transactivation domain and activates it by phosphorylating Ser-63 and Ser-73 [59]. JNK is expressed throughout neurons and is required in axogenesis. In young axons, activated JNK forms a proximodistal gradient of proteins starting from the primordium to targeted neurites [60]. Lufen Chang et al. demonstrated that JNK1, an isoform of JNK in mammals, is essential for maintaining the cytoskeletal integrity of neuronal cells by regulating microtubule assembly [61]. In the spinal cord, JNK is also involved in regulating axonal pathfinding processes of different neuronal subtypes, and mice with genetic deletion of JNK-pathway components show axonal anomalies [62]. Our study found that the *c-Jun* KO impaired NPC differentiation and observed impeded neuron axon growth.

Utilizing a high-resolution panoramic scanning system (TissueFAXS), we have acquired and analyzed batches of images from histological sections of mouse embryos, identifying losses of nerve fibers within the brain parenchyma and structural anomalies in the thalamus. These phenotypic discrepancies were replicated in both 2D neurons and 3D organoid models, which were established through both unguided and guided methods. Initially, we selected unguided COs as a model due to their higher structural diversity and the inclusion of various functional regions. Whole-brain models enabled us to capture these phenotypic differences early on, when the affected regions were not yet clearly defined. Clues from mouse embryos and COs suggested the vulnerability of thalamus regions in the diencephalon. Subsequently, we hypothesized that the thalamus might be the most susceptible to *c-Jun* loss and established a specific thalamus model through directional induction. This targeted thalamus model not only allows us to test the hypotheses emerging from mouse and whole-brain models but also facilitates experiments, such as rescue assays or omics sequencing analysis.

The thalamus, located in the diencephalon, serves as a crucial relay station facilitating communication between the cortex and lower nerve systems. Its development and maintenance heavily depend on the proper guidance and projection of nerve fibers [63, 64]. Cell–cell adhesion molecules also play a critical role in the fasciculation and routing of thalamocortical and corticothalamic fibers [65]. Given its protruding nature and reliance on intercellular connections, the thalamus in the diencephalon may be particularly susceptible to disturbances in fiber projection [64, 66]. A recent study has highlighted the necessity of *c-Jun* N-terminal kinase signaling in the early construction of the thalamocortical axon pathway within the ventral forebrain. Complete genetic removal of JNK signaling in mice hinders thalamocortical axons from crossing the diencephalon-telencephalon boundary and disrupts internal capsule formation [67]. Our data indicate that the *c-Jun* KO may affect the tight-junction integrity of cells. The loss of cell-cell connections may hinder the construction of the axon pathway. Moreover, our research observed that *c-Jun* KO impedes fiber extension in neurons, as well as in organoids with specific TCF7L2^+^ identity. If guidance and projection are compromised due to the loss of structural or connection proteins, the neuron maturation and migration would be affected, potentially leading to developmental abnormalities or loss of existing structures. This may elucidate why cells and fibers were scarce in KO neural models, and the contour of the thalamus was abnormal in the brains of KO mouse embryos. Another possible explanation is that *c-Jun* deficiency leads to increased neuronal apoptosis, which damages thalamus development. In the mouse model, we found increased signals of inflammatory and apoptosis/death in the damaged *c-Jun* KO forebrain and thalamus/diencephalon. We detected universal apoptotic signals but did not find significant difference between WT and KO in NPC as well as organoids models. Whether the apoptosis/death effect leads to the decrease in the volume and cell density of ThOs still needs further detailed investigation.

Due to potential variations in the time point of death in KO embryos [18, 19], some embryos may be affected by KO early on and fail to form a mature diencephalic thalamus, rendering corresponding signals (e.g., TCF7L2) undetectable. Conversely, embryos that perish later may experience limited KO influence, allowing for the formation of mature marker cells, albeit with affected expression and distribution compared to normal embryos. This variability could explain the different TCF7L2 staining patterns observed in KO embryos. The detail mechanism still needs further investigation in the future.

Our study observed the *c-Jun* KO promotes cortex development in COs, whereas, several cortical neurons genes are downregulated in *c-Jun* defected ThOs. CO model contains a variety of cell types, and ThO model contains mainly the thalamus specific cell types. GO analysis, therefore, may reflect the different cell types in different microenvironment. It is possible that in the unguided whole brain, groups of central nervous genes are up-regulated while in the guided thalamus induction, some of CNS genes are down regulated, especially they are related to telencephalon or hindbrain. We traced the 3 up-regulated genes (*PTPRZ1*, *SLC2A1*, and *POU3F3*) in ThO RNA-seq, which are classified as genes in central nervous system, and find that their distribution is mainly in forebrain, telencephalon, and hindbrain (data from MOSTA) [21] and in the midbrain in some reports [68]; It is also possible that homologues within the same catalog have complementary or even opposite functions, and show different trends of change. The deficiency of c-Jun may have broad effects on neurodevelopment and lead to a variety of neurodevelopmental disorders such as neuronal migration disorders, synapses deformity, and neurodegenerative diseases or mental illness. Future studies are needed to explore their features under *c-Jun* stimulation, which may depict a more comprehensive picture of *c-Jun* in central nervous system development.

The deep-seated location of the thalamus/diencephalon within the mammalian brain poses significant challenges in deciphering its development and plasticity. The linkage between *c-Jun* and thalamus development offers new insights into understanding the development and maintenance of this under-studied region in the CNS.

Overall, our findings uncover a novel role of *c-Jun* in neural development and suggest a vulnerable region within the CNS susceptible to damage when its normal regulatory function is disrupted. Given *c-Jun*’s central position in a vast gene regulatory network, further investigation into its roles promises to unravel far-reaching mysteries in the field of neuroscience.

## Materials and methods

### Animals

*c-Jun*^*+/−*^ mice were procured from the Guangzhou Institutes of Biomedicine and Health (GIBH), Chinese Academy of Sciences. *c-Jun*^*−/−*^ KO mice were developed using standard methods. The WT littermates (*c-Jun*^*+/+*^) were used as controls. All animals were maintained under the conditions used for C57BL/6J mice and housed at the animal facility center of GIBH. For embryo staging, the mice were allowed to mate in the afternoon and then separated the next morning. The day when they were separated at noon was considered embryonic day 0.5 (E0.5).

### Embryo processing and H&E staining

The embryos were removed and separated in cold phosphate-buffered saline (PBS). Then, they were immediately transferred to a 4% paraformaldehyde (PFA, Sigma-Aldrich) solution and incubated overnight at 4℃. The 4% PFA solution was changed the next day. Embryos were maintained in this soaking condition until use. Sagittal paraffin-embedded (5 μm) or frozen (10 μm) sections from the midline area of the brain were prepared. Standard protocols were used to perform H&E staining.

### DiI dying and diffusion assay

The mouse embryos fixed with 4% PFA were sliced into two equal parts along the sagittal direction and embedded with DiI crystal granules close to the diencephalon region. Then, the tissues were immersed in 1× PBS and placed in a 37℃ incubator in the dark. After static incubation for 1 week, the samples were visualized under bright light using a stereomicroscope.

### Cell culture

H1 ESCs and *c-JUN* KO H1 ESCs established using the CRISPR-Cas9 system were as previously described [12]. H1 ESCs were maintained in mTeSR1 medium (STEMCELL Technologies, Canada) on Matrigel-coated plates. Stem cells were routinely passaged with ethylenediaminetetraacetic acid (EDTA) every 4–5 days. Cells passage 65–75 were used for the experiments.

### Cytogenetic analysis

H1 ESCs were seeded onto diluted Matrigel-coated 6-well plates and cultured till 70–80% confluency. First, ESCs were treated with colcemid for 2 h at 37℃; then, cells were harvested by dispersing accutase for 3–5 min at 37℃. Standard protocols were used to prepare metaphase slides. A previously described method was used to perform chromosome analysis [69].

### NPC culture

NPCs were induced according to the protocol established by Shi et al. [70]. Briefly, dissocicated ESCs were resuspended in the neural maintenance medium (NMM). NMM contained a 1:1 mixture of DMEM/F-12 and neurobasal medium supplemented with 0.5% (v/v) N-2, 1% (v/v) B-27, 1% (v/v) GlutaMAX, 5 µg/mL insulin, 0.5% (v/v) nonessential amino acids, 0.5 mM sodium pyruvate, 50 µM 2-mercaptoethanol (1:100 dilution in 1× Dulbecco’s PBS [DPBS]), 50 U/mL penicillin, and 50 µg/mL streptomycin. The cells were plated at a density of 1–1.5 × 10^6^/well in a Matrigel-coated 12-well plate and cultured till they reached 100% confluency within 1 day after plating. Upon reaching 100% confluency, the medium was switched to NMM + 1 µM dorsomorphin + 10 µM SB431542 and refreshed daily from day 1 to day 11. On day 12, 5 U/mL dispase (Stemcell, 07913) was added. Subsequently, the rosette sheets were sliced into small square clumps of approximately 1–2 mm in size. These clumps were transferred into a new 12-well plate coated with poly-L-ornithine (Sigma, P4957) and laminin (Sigma, L2020) at a ratio of 1:2. From day 13 to day 17, the medium was switched to NMM + 20 ng/mL FGF-2 (Peprotech, 100-18B). FGF-2 was removed after day 17. On day 20, the cells were further passaged in the presence of dispase at a 1:2 ratio. At this point, cells were subjected to flow cytometry or other downstream analyses. Cells that remained could be cryopreserved on day 25. For long-time culture after day 25, the cells sheet was treated with dispase, followed by slicing the sheet into small clumps. Cells were passaged at a ratio of 1:2 in a laminin-coated 12-well plate. The medium was refreshed every other day.

### CO culture

The COs culture were established according to the Lancaster group’s protocol [24, 25] with adaptations. Briefly, 10,000 cells were suspended in embryoid body (EB) medium and seeded into 96-well U bottom plates (PrimeSurface, MS-9096UZ) for EB formation. The EB medium consisted of DMEM/F12 supplemented with 20% (v/v) KnockOut serum replacement, 3% (v/v) fetal bovine serum, 1% (v/v) nonessential amino acids, 1% (v/v) GlutaMAX, 100 µM 2-mercaptoethanol (1:100 dilution in 1× DPBS), 4 ng/mL basic fibroblast growth factor, and 50 µM Y-27,632 (for the first 4 days). After 6 days of aggregation, an EB with a diameter of 500–600 μm formed in each well and was transferred to neural induction medium for an extended period of 7 days. The neural induction medium comprised DMEM/F12 supplemented with 1% (v/v) N2, 1%(v/v) nonessential amino acids, 1% (v/v) GlutaMAX, and 1 µg/mL heparin. After induction for 7 days, a layer of transparent neuroectoderm appeared at the outer edge of the bulb, indicating successful nerve induction. Subsequently, the spheroids were embedded in Matrigel and transferred to 6-well plates containing neural differentiation medium (≤ 10 spheroids/well). The neural differentiation medium comprised a 1:1 mixture of DMEM/F-12 and neurobasal medium supplemented with 0.5% (v/v) N-2, 1% (v/v) B-27 (without vitamin A for the first 3 days), 0.5%(v/v) nonessential amino acids, 1% (v/v) GlutaMAX, 50 µM 2-mercaptoethanol (1:100 dilution in 1× DPBS), 5 µg/mL insulin, 50 U/mL penicillin, and 50 µg/mL streptomycin. Once the budding structure grew and enlarged under static conditions, the plates were placed on a horizontal shaker at 90 rpm for rotation and medium was refreshed every 3–4 days. Morphological changes were monitored and analyzed at specific intervals.

### ThO culture

ThOs cultures were established based on a published protocol [46, 47] with modifications. Briefly, 10,000 suspended single cells in EB Medium were seeded into 96-well U bottom plates (PrimeSurface, MS-9096UZ) for EB formation and neural induction. The EB medium comprised DMEM/F12 supplemented with 20% (v/v) KnockOut serum replacement, 1% (v/v) nonessential amino acids, 1%(v/v) GlutaMAX, 100 µM 2-mercaptoethanol (1:100 dilution in 1× DPBS), 5 µg/mL insulin, 100 nM LDN-193,189, 10 µM SB431542, and 50 µM Y-27,632 (for the first 4 days). On day 8, EBs with a diameter of approximately 500–600 μm were transferred to an ultra-low attachment 24-well plate with 1 mL thalamus patterning medium (TPM). Spinning culture was performed at a speed of 85 rpm for another 8 days to achieve thalamus fate. The TPM comprised DMEM/F12 supplemented with 0.15% (w/v) dextrose, 0.5% (v/v) N2, 1% (v/v) B27 without vitamin A, 30 ng/mL BMP7, and 1 µM PD325901. On day 16, the organoids were transferred into new ultra-low attachment 24-well plates comprising 1 mL of the neural differentiation medium, followed by a spinning culture at 85 rpm. The neural differentiation medium was based on the medium used in COs; and 20 ng/mL BDNF and 200 µM ascorbic acid were added. The neural differentiation medium was replenished every other day before day 25; thereafter, it was replaced every 4 days.

### Induction/over expression of *c-JUN*

The tet-on system was established through the plasmid transfection and the *c-JUN* induction or overexpression was induced via adding doxycycline (DOX) in culture medium. For ESCs and NPCs, the working concentration of DOX is 0.5 µg/mL and for organoids the working concentration is 2.5 µg/mL.

### Quantitative real-time PCR

TRIzol (Invitrogen, 15596026) was used to extract the total RNA. Then, 1 µg of total RNA was used to synthesize cDNA using the HiScript^®^II Q RT SuperMix for qPCR (+ gDNA wiper) (Vazyme, R223-01) according to the manufacturer’s instructions. Real-time PCR was conducted using the ChamQ SYBR qPCR Master Mix (Vazyme, Q311-02/03) and the ABI Step One Plus system (Applied Biosystems). Samples were run in triplicate and normalized to GAPDH expression. Relative expression was calculated using the ΔCT method. Table S1 lists the primer sequences.

### IF analysis

Cells were grown on coated confocal dishes. COs and mice embryo tissues were fixed with 4% PFA overnight and then dehydrated using a 30% sucrose gradient. The samples were embedded with OCT (Leica, FSC22) and frozen sections were prepared. Before IF staining, the samples were washed with PBS and fixed with 4% PFA for 15 min. After washing three times, the cells or sections were blocked with PBS containing 0.1% Triton-X 100 (Sigma, 1001843780) and 1% bovine serum albumin (BSA) (Biosharp, BS043E) at room temperature (RT, 25℃) for 1 h. Next, the cells or tissues were incubated with primary antibodies (Table S2) containing 0.1% Triton-X 100 and 1% BSA at 4℃ overnight. After washing three times with PBS, the samples were incubated with the secondary antibodies in PBS with Hoechst at RT for 1 h. After washing and sealing, then performed the imaging.

### Flow cytometry

To determine the differences in cell composition, the NPCs cultured in a poly-L-ornithine/laminin-coated 12-well cell culture dish were washed with 1× DPBS, dissociated with accutase, and additionally fixed and stained with primary and secondary antibodies based on the optimized operational processes. Finally, analysis was performed using FACS Fortessa (BD Biosciences). Table S2 presents the antibodies used.

### Western blotting

Whole-cell lysates were prepared by lysing the cell pellets in RIPA buffer comprising protease and phosphatase inhibitors, phenylmethylsulfonyl fluoride, and EDTA. The lysates were incubated for 10 min on ice and then subjected to three rounds of sonication for 5 s each time. The samples were centrifuged at 10,000 ×*g* and 4℃ for 20 min. The supernatant was collected. Protein concentration was measured using the BCA assay. Total protein was loaded onto a 12% acrylamide gel and then transferred to a nitrocellulose membrane using the Trans-Blot Turbo Transfer System (Bio-Rad, 1704150). Table S2 lists the antibodies used and their respective dilutions.

### Imaging

TissueFAXS multi-mode scanning platform was used for H&E staining and TissueFAXS Viewer was employed for subsequent analysis. The LSM800 confocal laser scanning system (Zeiss) was used to capture IF images. An inverted stereomicroscope equipped with a camera (Zeiss) was employed to visualize bright-field images.

### RNA-seq

Total RNA extraction was performed using TRIzol (Invitrogen, 15596026) reagent. Pooled CO samples (3–4) were lysed with TRIzol by pipetting on ice. RNA extraction was performed according to the manufacturer’s instructions. After which the concentration and purity of RNA were determined. To construct the library, the RNA was diluted to an appropriate starting concentration. VAHTS^®^ Total RNA-seq (H/M/R) Library Prep Kit for Illumina (Vazyme, NR603) and VAHTS^®^RNA Adapters set3 for Illumina (Vazyme, N809) were utilized to construct the libraries for sequencing according to the manufacturer’s instructions. Briefly, the RNA sample was first hybridized with the probe, followed by digesting the rRNA with RNase H. The probe was then digested with DNase I. The RNA was broken into fragments of 150–200 bp of insertions. Random primers were used to synthesize the first cDNA strand. The dUTP label was incorporated in the complementary strand. After dA-tailing and adapter ligation (VAHTS^®^RNA Adapters set3: N809 for Illumina), UDG enzymes were used to digest the dUTP-labeled second chain template. PCR was performed to amplify the library. After verifying the library quality, sequencing was performed on the NovaSeq 6000 S5 platform. The double-ended sequencing program (PE) was run to obtain 150-bp sequencing reads.

### CUT&Tag

The Hyperactive Universal CUT&Tag Assay Kit (Vazyme) was applied according to the manufacturer’s instructions to generate *c-JUN* CUT&Tag data. Three ThOs were collected and digested using accutase at 37℃ for 30 min and dispersed with a pipette. Then 50,000 cells were used for the CUT&Tag assay. pA-Tn5 transposase was used to cut the genome, incorporating a special adaptor sequence to construct the library. Quantitative PCR (qPCR) was performed to detect enrichment of the target site in the library. The c-JUN antibody (#60A8; Cell Signaling Technology) was used to capture the bound proteins.

### ATAC-seq

A previously described method was employed to perform ATAC-seq [50]. Briefly, three ThOs were collected and digested using accutase at 37℃ for 30 min and dispersed with a pipette. Approximately 50,000 cells were washed with 50 µL of cold PBS and resuspended in 50 µL of lysis buffer (10 mM Tris-HCl, pH 7.4, 10 mM NaCl, 3 mM MgCl_2_, and 0.2% [vol/vol] IGEPAL CA-630). The nuclear suspension was centrifuged at 500 ×*g* and 4 °C for 10 min. Then, 50 µL of the transposition reaction mix (25 µL of TD buffer, 2.5 µL of Tn5 transposase, and 22.5 µL of nuclease-free H_2_O) from the Nextera DNA library Preparation Kit (96 samples) (FC-121-1031; Illumina) was added. PCR was performed to amplify the samples, followed by incubation at 37 °C for 30 min. DNA isolation was performed using the MinElute Kit (QIAGEN). The ATAC-seq libraries were subjected to 12 cycles of amplification and subsequently sequenced on the NextSeq 500 sequencing platform with the NextSeq 500 High Output Kit v2 (150 cycles) (FC-404-2002; Illumina) according to the manufacturer’s instructions.

### Bioinformatics analysis

All RNA-seq data have been deposited in NCBI’s Gene Expression Omnibus (http://www.ncbi.nlm.nih.gov/geo/). Differential expression gene analysis (for a set of samples) and gene expression heat map analysis (advanced edition) were conducted online via applications developed by Anoroad’s Big Data cloud computing platform (https://www.solargenomics.com/), *p* < 0.05, fold change (FC) ≥ 2. GO analysis was conducted on the online DAVID platform (https://david.ncifcrf.gov/tools.jsp). Volcano map analysis and PCA were conducted using imageGP (https://www.bic.ac.cn/ImageGP/), utilizing absolute Log2 fold change values of 1 and an adjusted *p*-value threshold of 0.05. ATAC sequence and CUT&Tag data were opened and analyzed using Integrative Genomics Viewer (IGV) (version: 2.16.2).

### Image data processing and statistical analysis

ImageJ (Fiji) was used to measure the thickness of the neural ectoderm in COs and the area of the TUJ1 and TCF7L2 signals in the thalamic neurons. ImageJ (Fiji) was also employed to measure the mean of signal intensity of IF staining in the orgnoids sections. All experiments were performed using at least three separate batches. Statistical analysis were performed using GraphPad Prism 9.3.1. Unless otherwise specified, unpaired Student’s t-tests were conducted to assess statistical significance at *p* < 0.05 (*), *p* < 0.01(**), and *p* < 0.001(***).

## Electronic supplementary material

Below is the link to the electronic supplementary material.

Supplementary Material 1

## Data Availability

All raw and processed RNA-seq data have been deposited to the NCBI GEO and are publicly available as of the date of publication with the accession numbers GSE243735 and GSE260732. Further information and requests for resources and reagents should be directed to and will be fulfilled by the corresponding authors Dongwei Li (lidongwei@gzhmu.edu.cn), Huanxing Su (huanxingsu@um.edu.mo), Dajiang Qin (qin_dajiang@gzhmu.edu.cn).
