# Supplementary material for: Impact of c-JUN deficiency on thalamus development in mice and human neural models
Source: Cell Biosci. 2024 Dec 20;14:149. doi: 10.1186/s13578-024-01303-8 (PMC11662577; doi:10.1186/s13578-024-01303-8)
Supplement: Supplementary file 1 — Supplementary Material 1 [file 13578_2024_1303_MOESM1_ESM.pdf]

Supplementary Figures and Tables

Impact of *c-JUN* Deficiency on Thalamus Development in Mice and Human Neural Models

Jiantao Shi, Qing Chen, Jianheng Lai, Jieying Zhu, Ran Zhang, Md. Abdul Mazid, Dongwei Li*, Huanxing Su*, Dajiang Qin*

***Correspondence:** lidongwei@gzhmu.edu.cn, huanxingsu@um.edu.mo, qin_dajiang@gzhmu.edu.cn

Fig. S1, related to Fig. 1

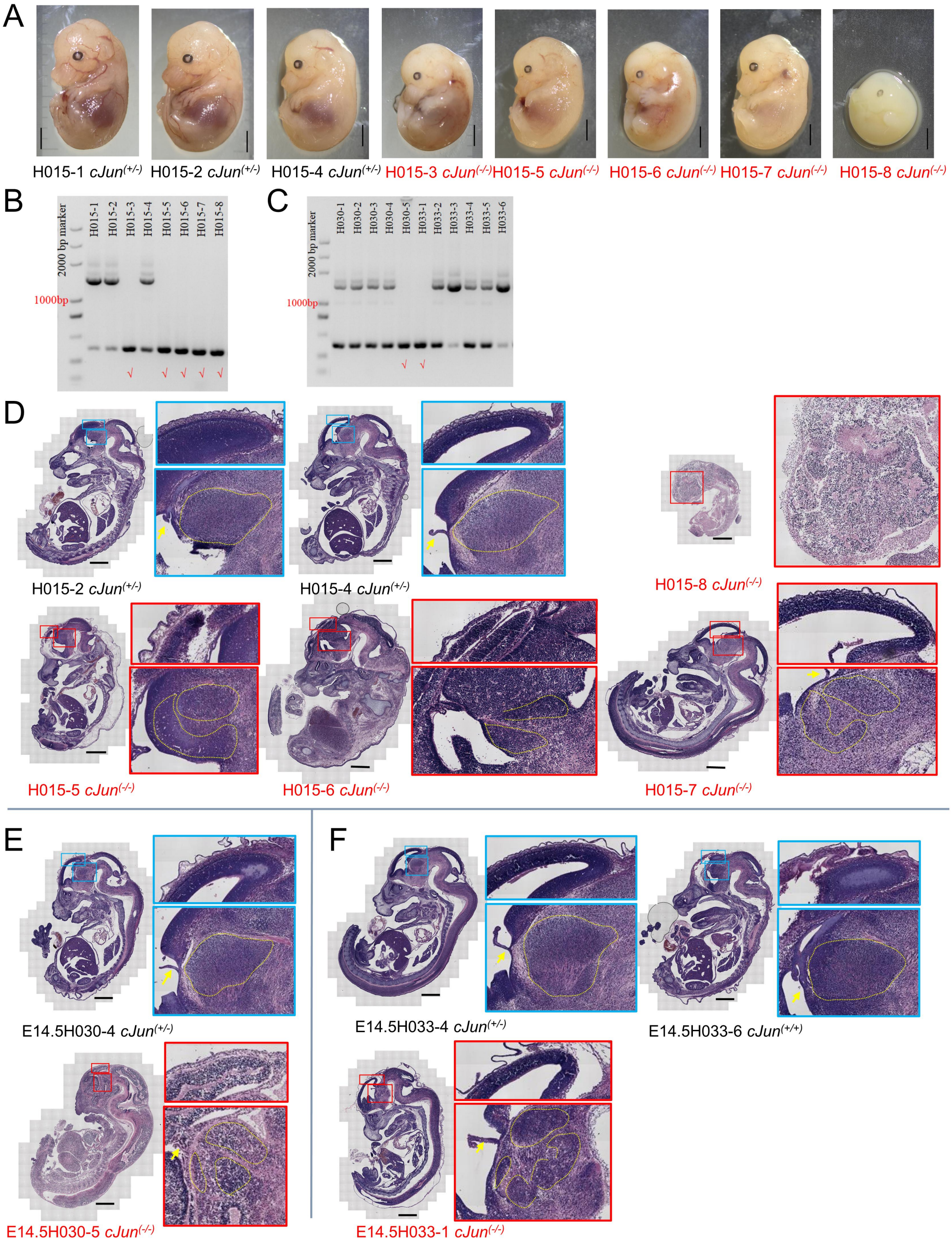

Figure S1. Thalamic malformation in diencephalon of KO mouse embryos at E14.5. Related to Figure 1. (A) BF images of 8 embryos at E14.5 from the litter H015 (self-defined code number of the mother mouse). BF, bright field. (B, C) Genotypes of the embryos in litters H015, H030 and H033. The red check marks indicate the KO genotype. (D) H&E staining of embryo H015 litter. Normal thalamus in WT (n=2, all heterozygote) and malformation thalamus in KO (n=4, all homozygote) embryos (circled with yellow dot line) were observed in litter H015 at E14.5. (E) Normal thalamus in WT (n=1, heterozygote) and malformation thalamus in KO (n=1, homozygote) embryos were observed in litter H030 at E14.5. (F) Normal thalamus in WT (n=2, 1homozygote, 1 heterozygote) and malformation thalamus in KO (n=1, homozygote) embryos were observed in litter H033 at E14.5. The yellow arrows in D-F indicated the choroid plexus, which serves as a position indicator to ensure the comparison of similar regions. Scale bar: 2000μm for (A); 1000μm for (D-F).

Fig. S2 related to Fig. 1

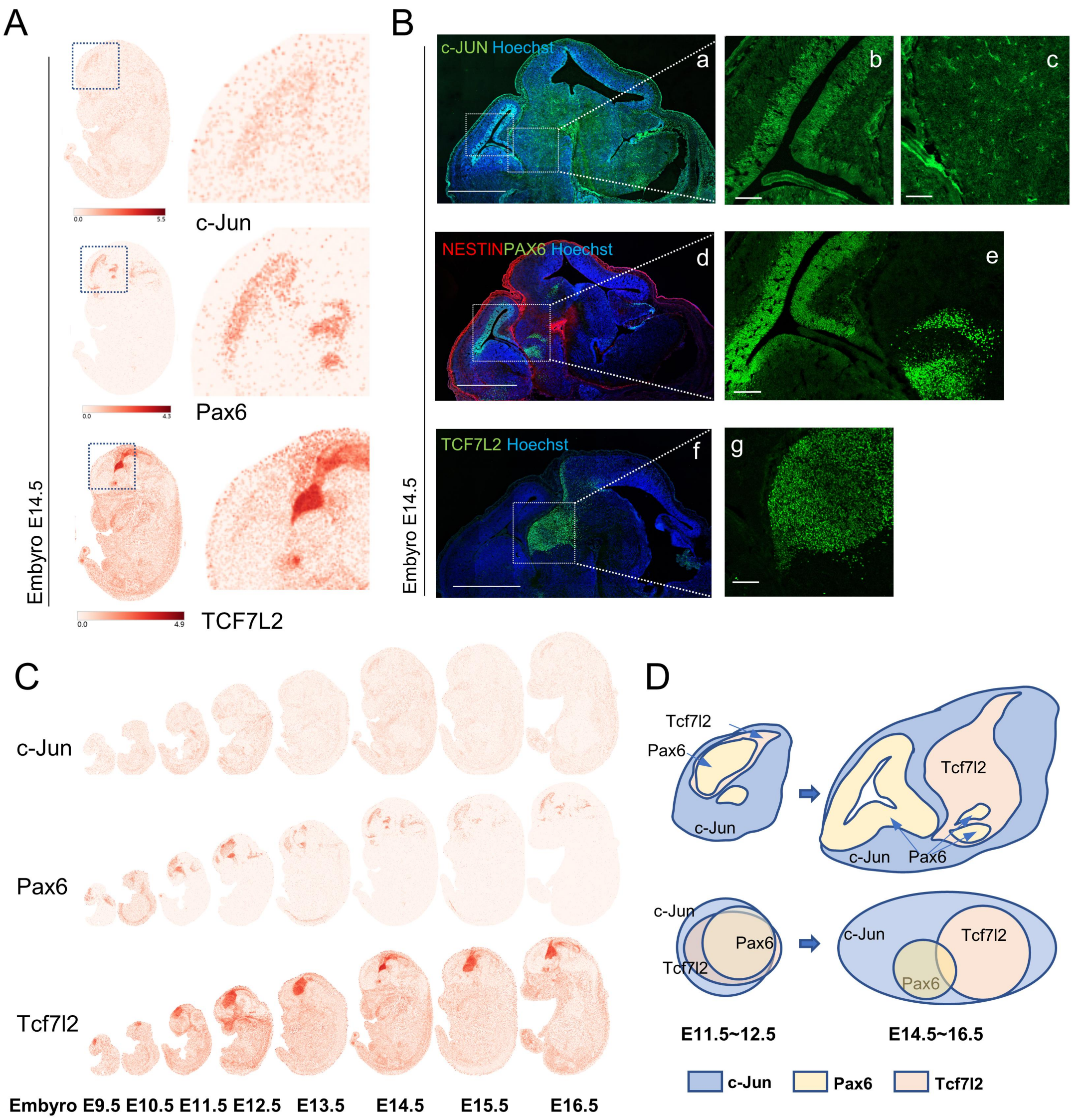

Figure S2. Spatiotemporal transcriptome analysis of *c-Jun*, *Pax6* and *Tcf7l2* during mouse embryonic development. Related to Figure 1. (A) Spatiotemporal transcriptome map of *c-Jun*, *Pax6* and *Tcf7l2* in mouse embryonic development at E14.5. Data downloaded from Mouse Organogenesis Spatiotemporal Transcriptomic Atlas (MOSTA), https://db.cngb.org/stomics/mosta/. (B) IF analysis of embryonic CNS at E14.5 confirmed the expression patterns of 3 genes in the MOSTA in A. Scale bar: 1000μm for a, d, f; 100μm for b, c, e, g. (C) A continued spatiotemporal transcriptome map of *c-Jun*, *Pax6* and *Tcf7l2* in mouse embryonic development from E9.5 to E16.5. Data downloaded from MOSTA. (D) Diagrams of 3 genes expression location in CNS of mouse embryo (upper panel) and simplified relationship (lower panel) in two stages based on MOSTA. The expression sites of *c-Jun*, *Pax6* and *Tcf7l2* generally overlapped in the earlier stage E9.5~E12.5 in CNS and separated in the later stage E14.5~E16.5, but *c-Jun* still partially overlapped with the other 2 genes in the later stage.

Fig. S3 related to Fig. 2

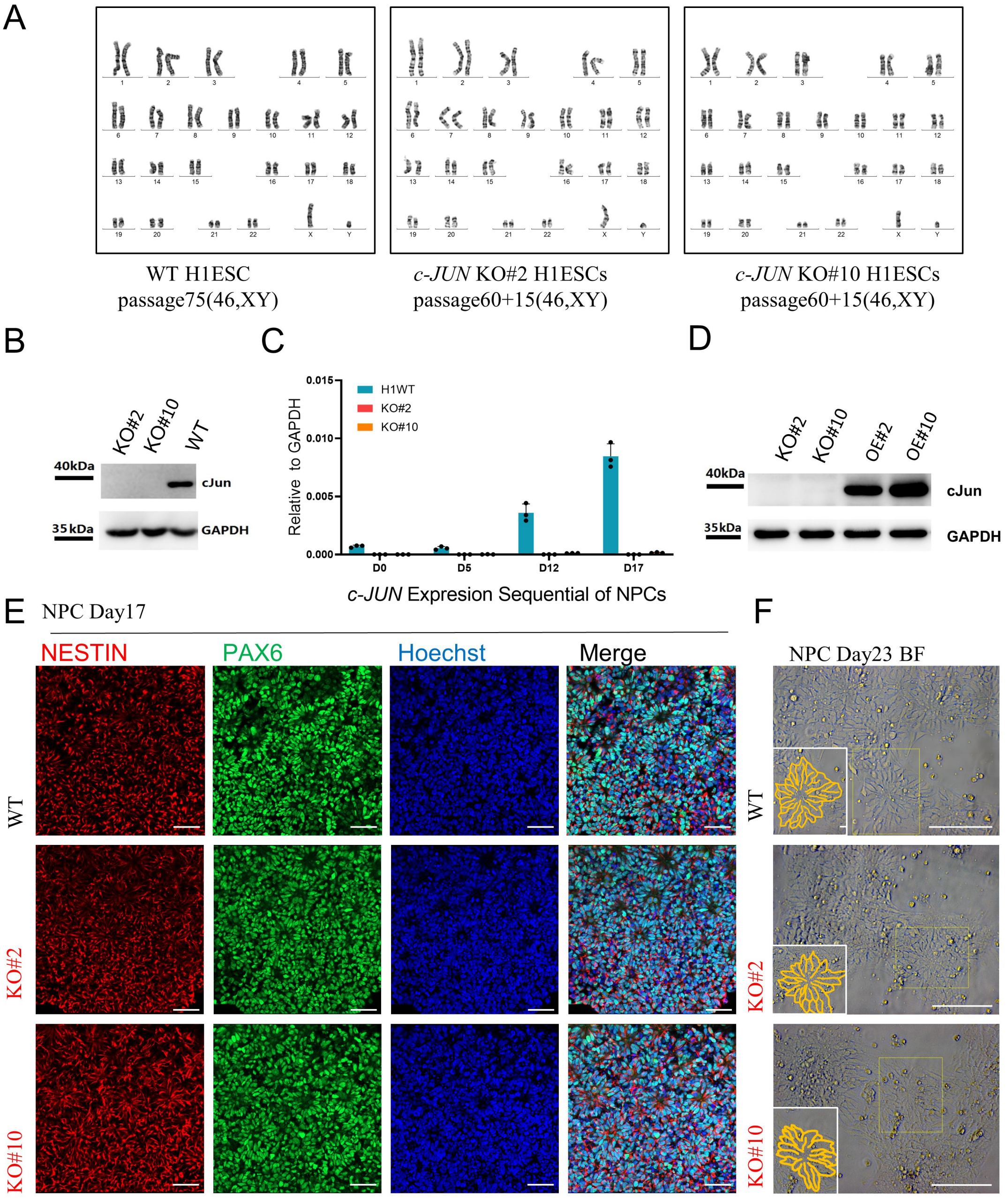

Figure S3. Normal rosette structures were observed in both WT and KO derived NPC. Related to Figure 2. (A) Karyotype analysis of WT (H1ESCs) and KO (H1ESC *c-JUN* KO#2, #10) show normal karyotype in all three cell lines. (B) Western blot analysis confirmed the null expression of c-JUN protein in KO cell lines. (C) RT-qPCR validation revealed the absence of *c-JUN* expression in KO groups during NPCs induction. (D) Western blot analysis confirmed the overexpression of c-JUN induced by DOX via the tet-on system. (E) IF staining of the NPC markers PAX6 and NESTIN on Day17 showed that both WT and KO derived NPC could form $PAX6^{+}$/$NESTIN^{+}$ neural rosettes. Scale bar: 50μm. (F) Typical rosettes were observed on day 23 under bright-light microscope. One representative rosette from each group was circled with yellow line to highlight the structure. BF: bright-light field; Scale bar: 50μm.

Fig. S4 related to Fig. 4

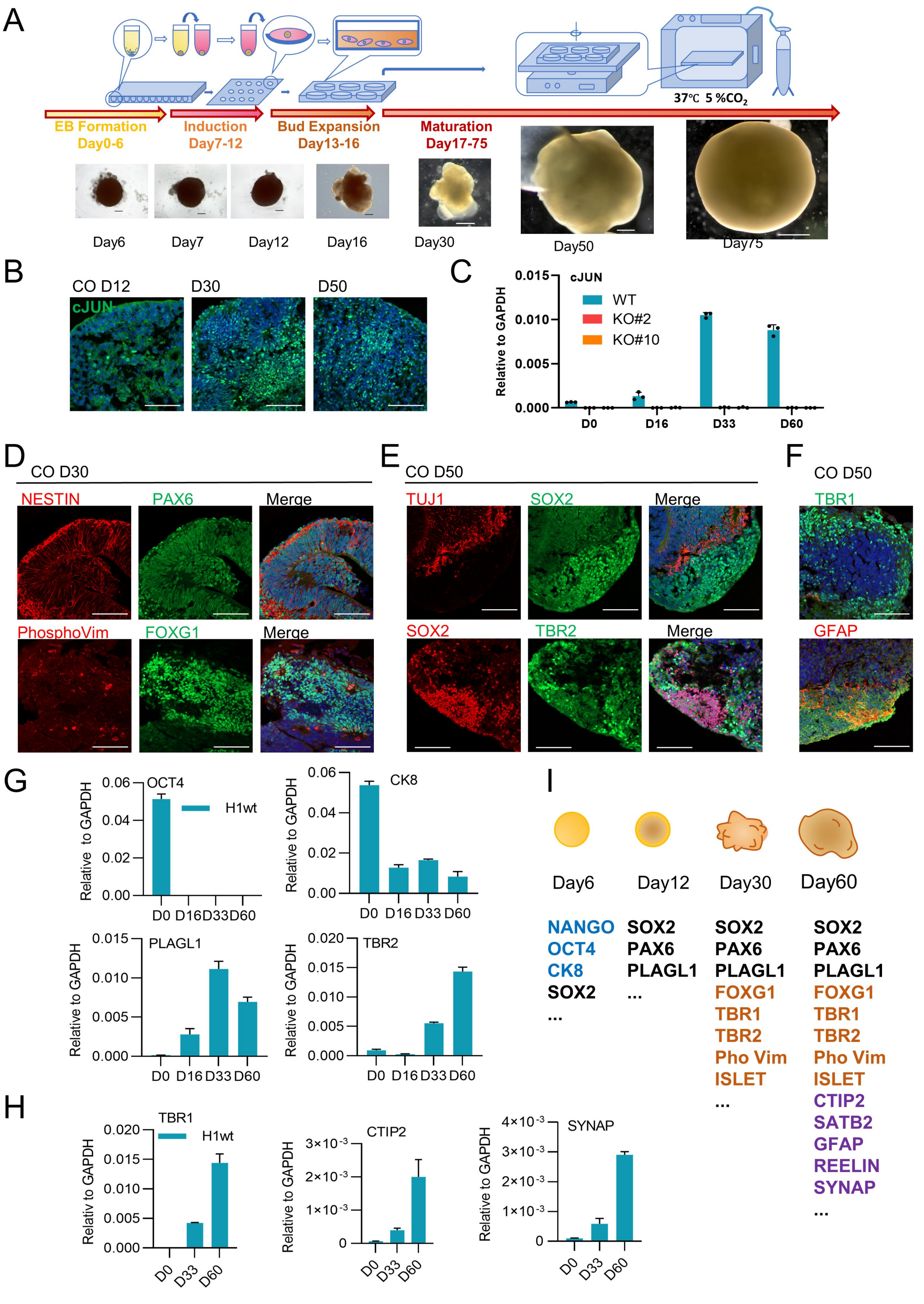

Figure S4. Induction and characterization of CO. Related to Figure 4. (A) Schematic of CO induction and global overview of CO in bright field. Scale bar: 200μm for day 6-16; 500μm for day 30-75. (B) IF staining of c-JUN in CO sections at day 12, 30 and 50. Scale bar: 100μm. (C) RT-qPCR analysis of *c-Jun* in CO at four time points (3 independent experiments). KO groups show null expression of *c-Jun*. Values are presented as mean ± SD. (D-F) IF staining of CO with several feature markers. Scale bar: 100μm. (G) RT-qPCR analysis of several stem cell and neural marker genes (3 independent experiments). (H) RT-qPCR analysis of late-expression cortical plate and synaptic function marker genes (3 independent experiments). Values are presented as mean ± SD. (I) A brief summary of gene expression sequence diagram in CO.

Fig. S5 related to Fig. 7

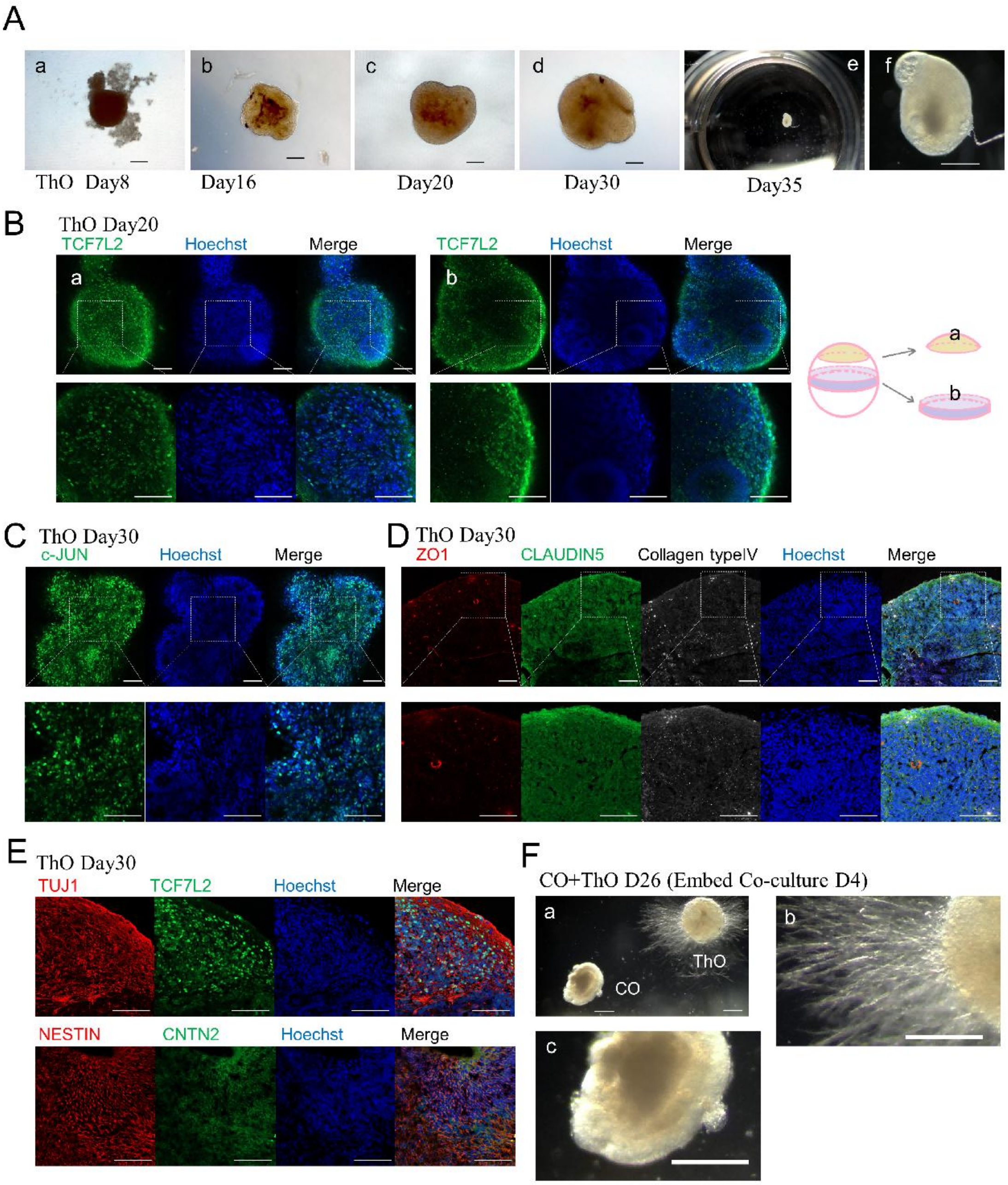

Figure S5 ThO induction and characterization. Related to Figure 7. (A) Global overview in bright field of ThO induction from H1ESC cell lines from day 8 to day 35. Scale bar: 200μm for a-d; 500μm for f. (B) IF staining of ThO on day 20 showed TCF7L2^{+} cells on upper sphere (a) and internal sphere (b). Scale bar: 100μm. (C) IF staining of ThO on day 30 showed c-JUN expressed ubiquitously. Scale bar: 100μm. (D, E) IF staining of ThO on day 30. The fibra-like structure in ThO showed signals of ZO1, CLAUDIN5, Collagen type IV, TUJ1, NESTIN and CNTN2 on day 30. Scale bar: 100μm. (G) Global overview in bright field of Matrigel embeded CO and ThO on day 26 (co-culture day 4), a: CO+ThO, b: ThO, c: CO. Scale bar: 500μm.

Table S1. Mouse embryos collected in the study

Age	Litters	Total embryos	c-Jun KO embryos	Percent of KO embryos
E11.5	4	32	10	31.25%
E12.5	5	44	12	27.27%
E13.5	6	41	9	21.95%
E14.5	15	121	26	21.49%

Table S2. Oligonucleotide sequences used for qRT-PCR

Gene	Sequence 5’ → 3’
h-GAPDH-F	TGACATCAAGAAGGTGGTGAAGCAGG
h-GAPDH-R	GCGTCAAAGGTGGAGGAGTGGGT
h-OCT4-F	CTGGGTTGATCCTCGGACCT
h-OCT4-R	CCATCGGAGTTGCTCTCCA
h-CK8-F	GAAGGGCTGACCGACGAGATCAA
h-CK8-R	CCAGCCAGGCTCTGCAGCTCC
h-ZO1-F	CAACATACAGTGACGCTTCACA
h-ZO1-R	CACTATTGACGTTTCCCCACTC
h-PLAGL1-F	AAAGATGCTTCTACACCCGGA
h-PLAGL1-R	AAAGATGCTTCTACACCCGGA
h-LMO3-F	GACACCAAGCCGAAAGGTTG
h-LMO3-R	ATGCCAGTATTTGTCCAGTGC
h-TBR1-F	GCAGCAGCTACCCACATTCA
h-TBR1-R	AGGTTGTCAGTGGTCGAGATA
h-TBR2-F	GTGCCCACGTCTACCTGTG
h-TBR2-R	CCTGCCCTGTTTCGTAATGAT
h-CTIP2-F	GGTGCCTGCTATGACAAGG
h-CTIP2-R	GGCTCGGACACTTTCCTGAG
h-SYNA-F	CTCGGCTTTGTGAAGGTGCT
h-SYNA-R	CTGAGGTCACTCTCGGTCTTG

**h-FOXG1-F**	**TGGGAGATAGGAAAGAGGTGAAAA**
**h-FOXG1-R**	**GCACCAGGCTGTTGATGCT**
**h-cJUN-F**	**CGAGTTCTGAGCTTTCAAGGT**
**h-cJUN-R**	**CGAGTTCTGAGCTTTCAAGGT**

Table S3. Primary antibodies used in this study

Host	Antigen	Concentration	Company	Product number
Rabbit	Pax6	1:200	Biolegend	901301
Rabbit	Sox2	1:200	Invitrogen	PA1-094
Rabbit	Tbr1	1:200	Abcam	Ab31940
Rabbit	Tbr2	1:200	Abcam	Ab9618
Rabbit	Foxg1	1:200	abcam	Ab18259
Rabbit	c-Jun	1:500	CST	60A8
Rabbit	TCF7L2	1:500	CST	2569T
Rabbit	Claudin5	1:500	Affbiotech	AF5216
Rabbit	CNTN2	1:500	Cozmo-Lab	PD28358
Mouse	ZO1	1:500	invitrogen	33-9100
Mouse	Tuj1	1:500	Covance	801201
Mouse	Phospho-Vim	1:200	MBLInternational	D076-3S
Mouse	Sox2	1:500	Abcam	Ab171380
Mouse	GFAP	1:200	sigma	G3893
Mouse	Nestin	1:500	CST	33475
